# Synopsis of Ecuadorian *Pterichis* (Orchidaceae)

**DOI:** 10.7717/peerj.10807

**Published:** 2021-02-09

**Authors:** Marta Kolanowska, Dariusz L. Szlachetko, Sławomir Nowak

**Affiliations:** 1 Department of Geobotany and Plant Ecology, Faculty of Biology and Environmental Protection, University of Lodz, Lodz, Poland; 2 Department of Biodiversity Research, Global Change Research Institute AS CR, Brno, Czech Republic; 3 Department of Plant Taxonomy and Nature Conservation, Faculty of Biology, University of Gdansk, Gdańsk, Poland

**Keywords:** Biodiversity, Cranichideae, Neotropics, New species, Taxonomy, Distribution

## Abstract

A taxonomic synopsis of the orchid genus *Pterichis* in Ecuador is presented. All national representatives of this genus are characterized and their floral segments are illustrated. Four new species and two new varieties are described. An updated key to Ecuadorian *Pterichis* is provided. Plants of the genus are growing mostly as terrestrial herbs at the altitude of 2,300–4,110 m. Often two or more species co-occur in the area of 25 × 25 km. Their occurrence was reported from three ecoregions—the Eastern Cordillera real montane forests, the Northern Andean páramo and the Northwestern Andean montane forests. Seven Ecuadorian *Pterichis* are endemic.

## Introduction

Amazonia and the Andes are some of the richest diversity hotspots in the world (Pennington et al., 2010; Antonelli et al., 2018). An extraordinary number of rare and endemic species is observed especially in the area where the two regions meet—the Tropical Andes. This hotspot extends from western Venezuela to northern Argentina and Chile, and incorporates significant part of territories of Colombia, Ecuador, Peru, and Bolivia. At the same time this is one of the most endangered ecoregions on the planet (Mittermeier et al., 1999), with a large portion of its landscape having been transformed. The fertile inter-Andean valleys of Colombia and Ecuador, are the most degraded as a result of agriculture and urbanization (Zador, 2015). The further habitat loss constitute a serious threat to numerous orchid genera (Hirtz, 2011).

Recognizing species composition is essential to preserve wildlife by identifying areas important for biodiversity and ecosystem services (Sofo, 2011; Casetta, Marques da Silva & Vecchi, 2019). For that reason, taxonomists are continuously publishing articles not only introducing the novelties in local biota, but also more comprehensive articles presenting concepts of particular plant or animal group (Ormerod, 2018; Damián Parizaca & Torres Montenegro, 2018). Orchidaceae and Asteraceae, with a rate of 500 new species being described each year (Chase et al., 2015), are among the most problematic groups to work with.

While globally about 2/3 of Orchidaceae are epiphytic or lithophytic plants, according to the International Union for Conservation of Nature almost half of the extinct orchids are terrestrial herbs (Swarts & Dixon, 2009). Unfortunately, no assessment of threats to tropical terrestrial/lithophytic orchids was done so far. The evaluation of the diversity of these plants and providing tools useful for their identification is essential to recognize the most valuable and biodiverse areas and to undertake any conservation actions.

Here we are dealing with the diversity of the mostly terrestrial genus *Pterichis* in Ecuador—one of the most orchid-rich countries in the Tropical Andes (Dodson, 2003; Meisel & Woodward, 2005). Despite the relatively long history of studies on the Ecuadorian Orchidaceae flora (Garay, 1978; Dodson & Dodson, 1980–1989; Dodson & Luer, 2005; Dodson & Luer, 2010), novelties are reported from this country each year (Zambrano Romero & Solano, 2019; Hágsater & Santiago, 2019).

As currently recognized, *Pterichis* encompasses ca. 45 species distributed from Costa Rica and Jamaica to Bolivia with the greatest diversity observed in Colombia (Kolanowska & Szlachetko, 2017). This genus was proposed in 1840 by Lindley to accommodate plants characterized by a mix of features of Cranichideae (helmet-shaped lip) and Spirantheae (plant habit). Representatives of *Pterichis* are usually terrestrial herbs with tuberous, clustered roots. Their scape is loosely sheathed and leaves are rosulate and often absent during flowering. The flowers are non-resupinate and arranged in a loosely to densely flowered raceme. Their sessile, concave, fleshy lip is usually densely papillate or/and ornamented with a series of swollen, knob-like cells along the margins (Kolanowska & Szlachetko, 2017).

Garay (1978) reported the occurrence of six *Pterichis* species from Ecuador considering *P. seleniglossa* as synonym of *P. triloba*, *P. barbifrons* as conspecific with *P. parvifolia* and *P. costaricensis* as synonym of P. *habenarioides*. In the last few years four additional species were described from this country (Szlachetko & Kolanowska, 2014; Kolanowska & Szlachetko, 2015; Kolanowska & Szlachetko, 2019).

The generic separateness of *Pterichis* has never been argued. The genus is clearly defined by its morphological characters—i.a. non-resupinate flowers with sessile, very broad lip which is concave in the center, variously ornamented with papillae or knob-like appendages. The monophyletic character of *Pterichis* was confirmed in the molecular studies (Salazar et al., 2003, 2009) and taxonomists agree with its placement in Cranichideae (Dressler, 1981; Dressler, 1993; Szlachetko, 1995; Chase et al., 2015).

The aim of this study was to evaluate the diversity of *Pterichis* species occurring in Ecuador, to provide key for their identification and to discuss the most discriminative morphological characters of each species.

## Materials and Methods

*Pterichis* specimens deposited in Ecuadorian herbaria HA, LOJA, Q, QAP, QCA, QCNE, and QPLS were examined in addition to material from AAU, AMES, COL, CUVC, F, FLAS, FMB, G, HUA, K, LPB, MO, NY, P, PSO, RPSC, UGDA, VALLE, and W. Specimens collected outside Ecuador were used as reference material to evaluate diagnostic usefulness of particular morphological features. Specimens were examined during visits in almost all of these facilities. Only material from AAU was loaned. Herbaria acronyms used in this article follow Thiers (2020). A total of over 100 Ecuadorian collections of *Pterichis* were examined.

The form of the leaf (if present) as well as the length and the surface of the scape were studied in the beginning. The assessment of the vegetative structures included also the tubular sheaths on the scape and the elements of the inflorescence (e.g., form of the floral bracts and ovaries). The perianth segments were observed after softening flowers in boiling water using a stereoscopic microscope. A database of original diagnoses and illustrations published by authors of particular taxa was created.

Morphological features presented in this study were compiled based on Ecuadorian material. The information provided on herbarium specimens labels was used for georeferencing. Distribution maps were prepared using ArcGIS 9.3 (Esri, Redlands, CA, USA). Species richness was calculated using DIVA-GIS (Hijmans et al., 2001). The concept of ecoregions follows Olson et al. (2001).

### Nomenclature

The electronic version of this article in portable document format will represent a published work according to the International Code of Nomenclature for algae, fungi, and plants (Turland et al., 2018), and hence the new names contained in the electronic version are effectively published under that Code from the electronic edition alone. In addition, new names contained in this work that have been issued with identifiers by IPNI will eventually be made available to the Global Names Index. The IPNI Life Science Identifiers (LSIDs) can be resolved and the associated information viewed through any standard web browser by appending the LSID contained in this publication to the prefix “http://ipni.org/.” The online version of this work is archived and available from the following digital repositories: PeerJ, PubMed Central, and CLOCKSS.

## Results

We confirmed 17 *Pterichis* species in Ecuador. Seven taxa, including four new species described here, are endemic—*P. ansaloniana, P. dodsoniana, P. elliptica, P. hirtziana, P. madsenii, P. meirax*, and *P. tunguraguona*.

**Figure 1:**
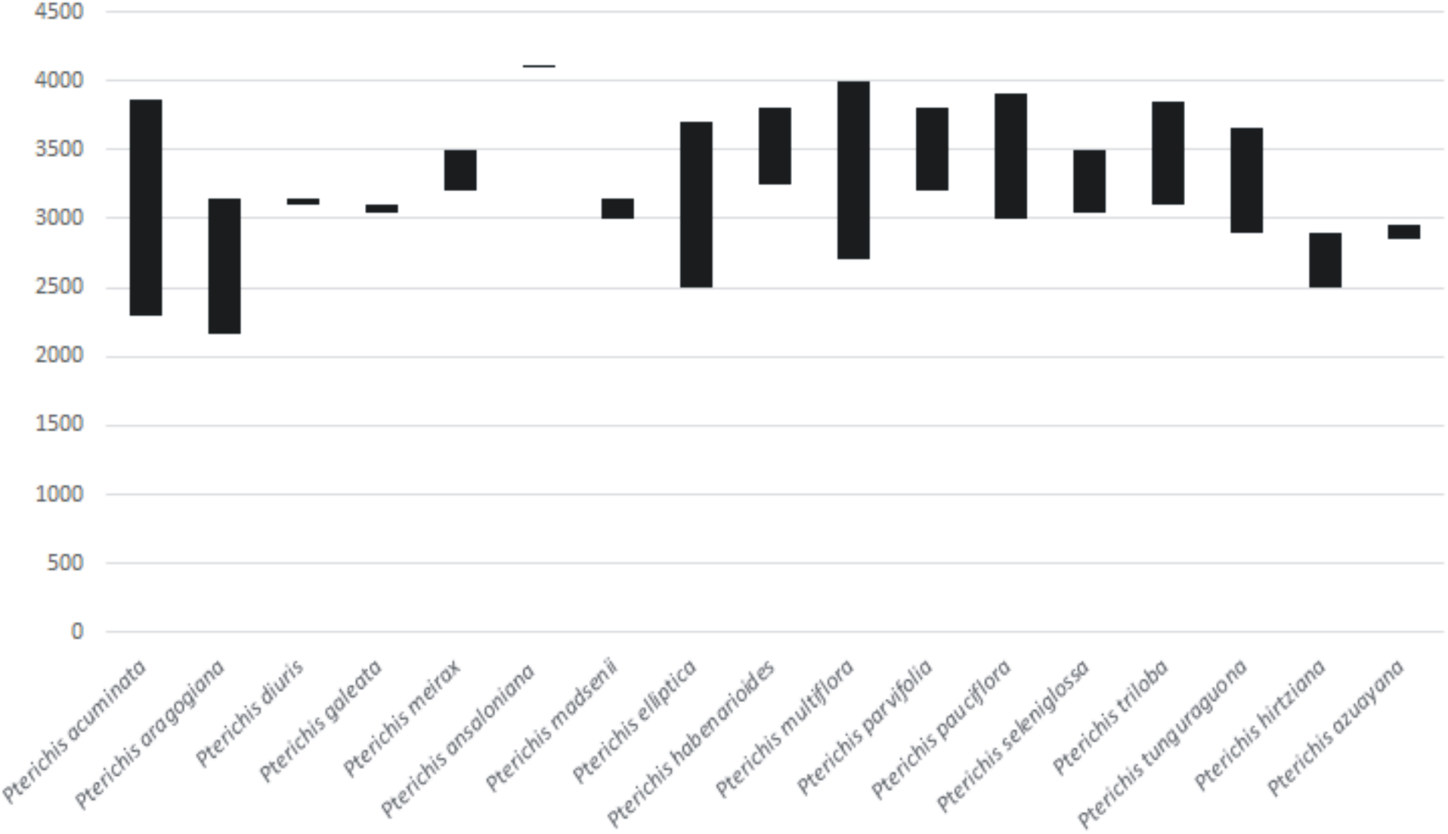
Altitudinal ranges of Ecuadorian *Pterichis*. Chart prepared in Microsoft Excel.

They are growing almost always as terrestrial herbs on roadsides, in cloud forest, humid montane forest and paramo at the altitudes of 2,300–4,110 m (Fig. 1). The broadest altitudinal range is observed in *P. acuminata* (2,300–3,870 m), *P. multiflora* (2,700–4,000 m), and *P. elliptica* (2,500–3,700 m).

**Figure 2:**
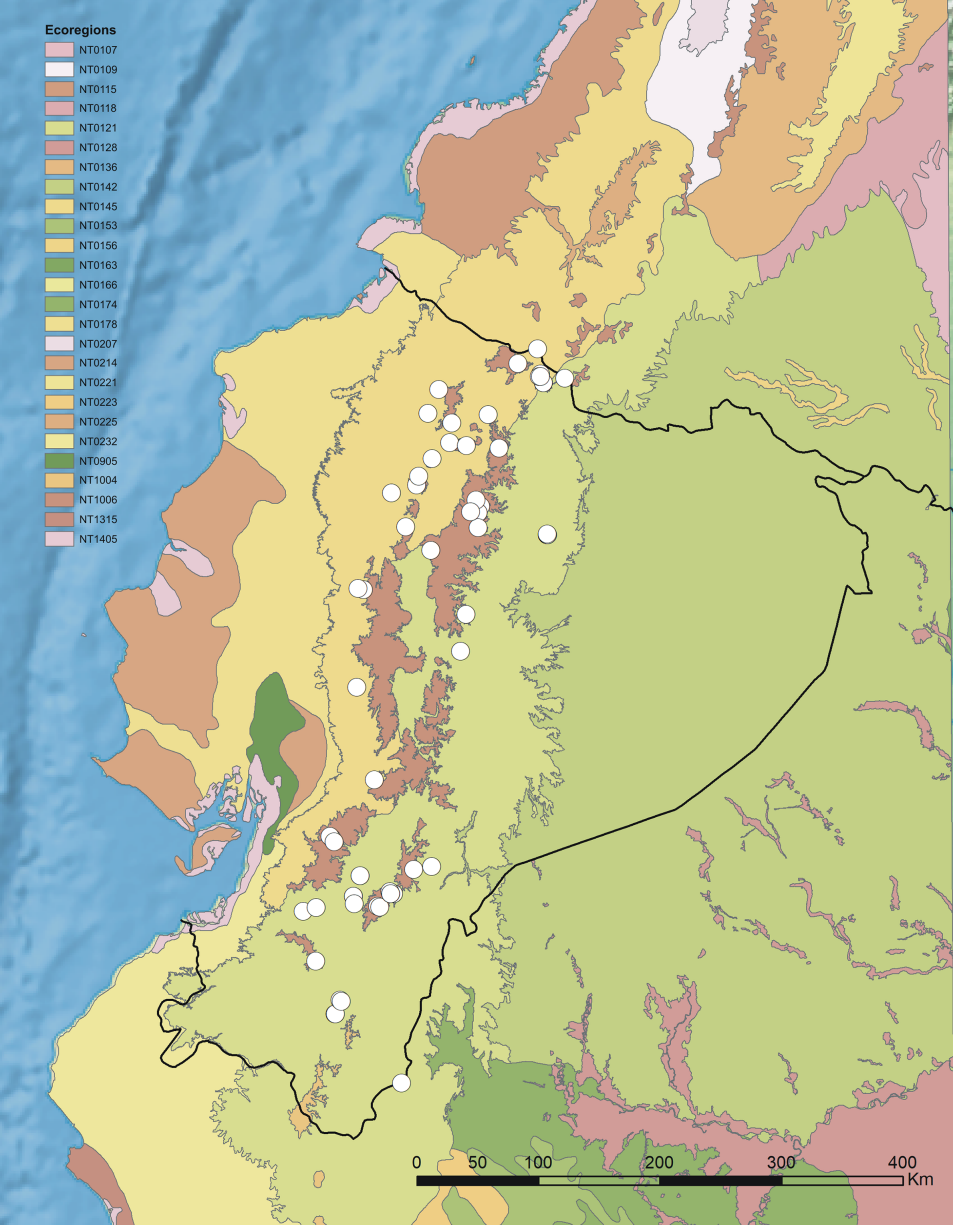
Terrestrial ecoregions of Ecuador with location of *Pterichis* populations. Ecoregion codes: NT0107—Caqueta moist forests, NT0109—Cauca Valley montane forests, NT0115—Chocó-Darién moist forests, NT0118—Cordillera Oriental montane forests, NT0121—Eastern Cordillera real montane forests, NT0128—Iquitos várzea, NT0136—Magdalena Valley montane forests, NT0142—Napo moist forests, NT0145—Northwestern Andean montane forests, NT0153—Peruvian Yungas, NT0156—Purus várzea, NT0163—Solimões-Japurá moist forests, NT0166—Southwest Amazon moist forests, NT0174—Ucayali moist forests, NT0178—Western Ecuador moist forests, NT0207—Cauca Valley dry forests, NT0214—Ecuadorian dry forests, NT0221—Magdalena Valley dry forests, NT0223—Marañón dry forests, NT0225—Patía Valley dry forests, NT0232—Tumbes-Piura dry forests, NT0905—Guayaquil flooded grasslands, NT1004—Cordillera Central páramo, NT1006—Northern Andean páramo, NT1315—Sechura desert, NT1405—South American Pacific mangroves. Map prepared in ArcGIS 9.3 (Esri, Redlands, CA, USA).

**Figure 3:**
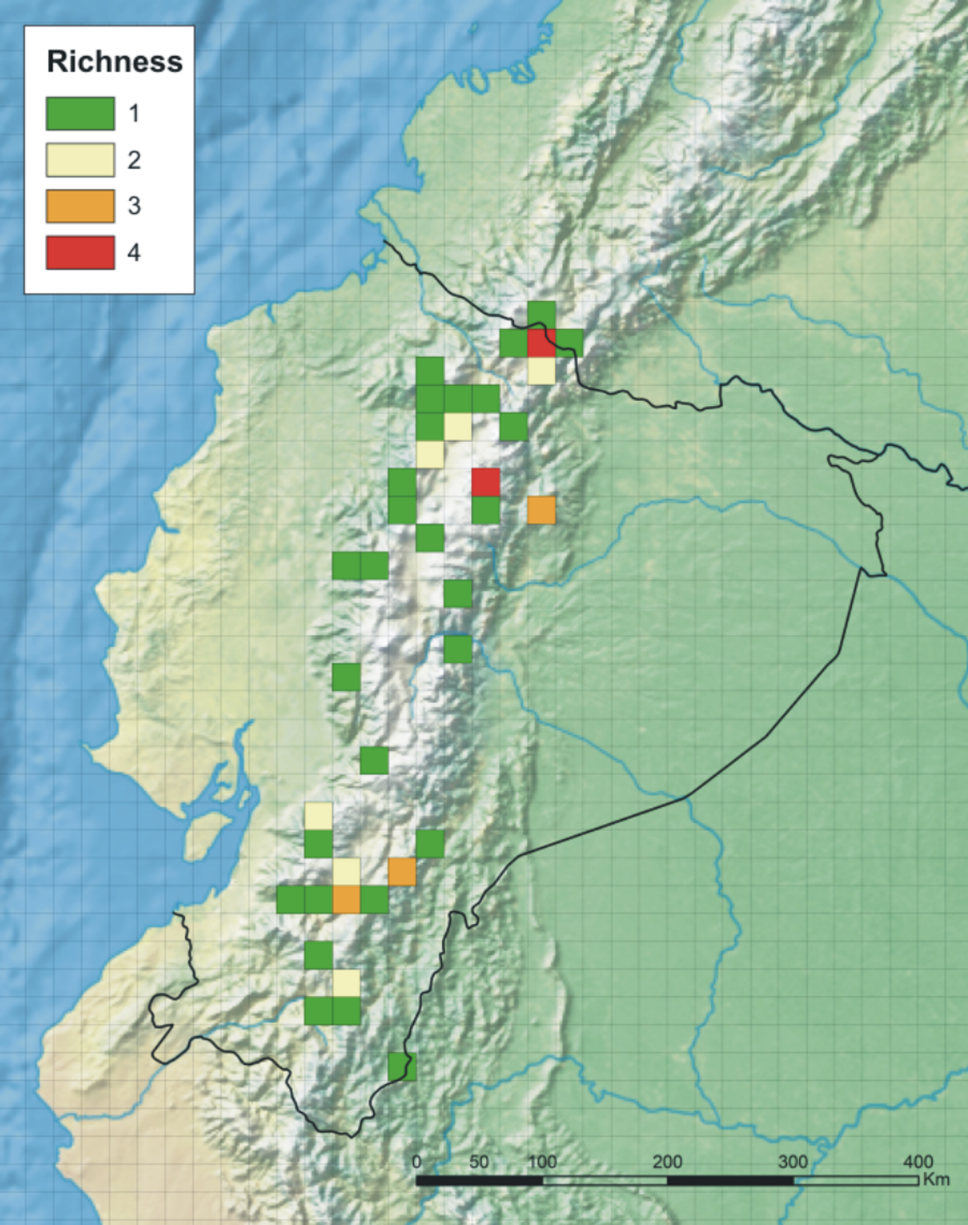
Richness of *Pterichis* species in Ecuador. Map prepared in ArcGIS 9.3 (Esri, Redlands, CA, USA) using outcomes of species richness analysis conducted in DIVA-GIS.

Ecuadorian *Pterichis* are distributed along the Andes in three various ecoregions (Olson et al., 2001; Fig. 2) and often two or more species co-occur in the area of 25 × 25 km (Fig. 3). The highest number of genus representatives (13) was found growing in the Eastern Cordillera real montane forests ecoregion (NT0121). Northern Andean páramo (NT1006) is a home to ten *Pterichis* species and seven taxa occur in Northwestern Andean montane forests (NT0145).

### Taxonomic treatment

***Pterichis*** Lindl., Gen. Sp. Orchid. Pl.: 444. 1840; Generitype: *Pterichis galeata* Lindl.

**Figure 4:**
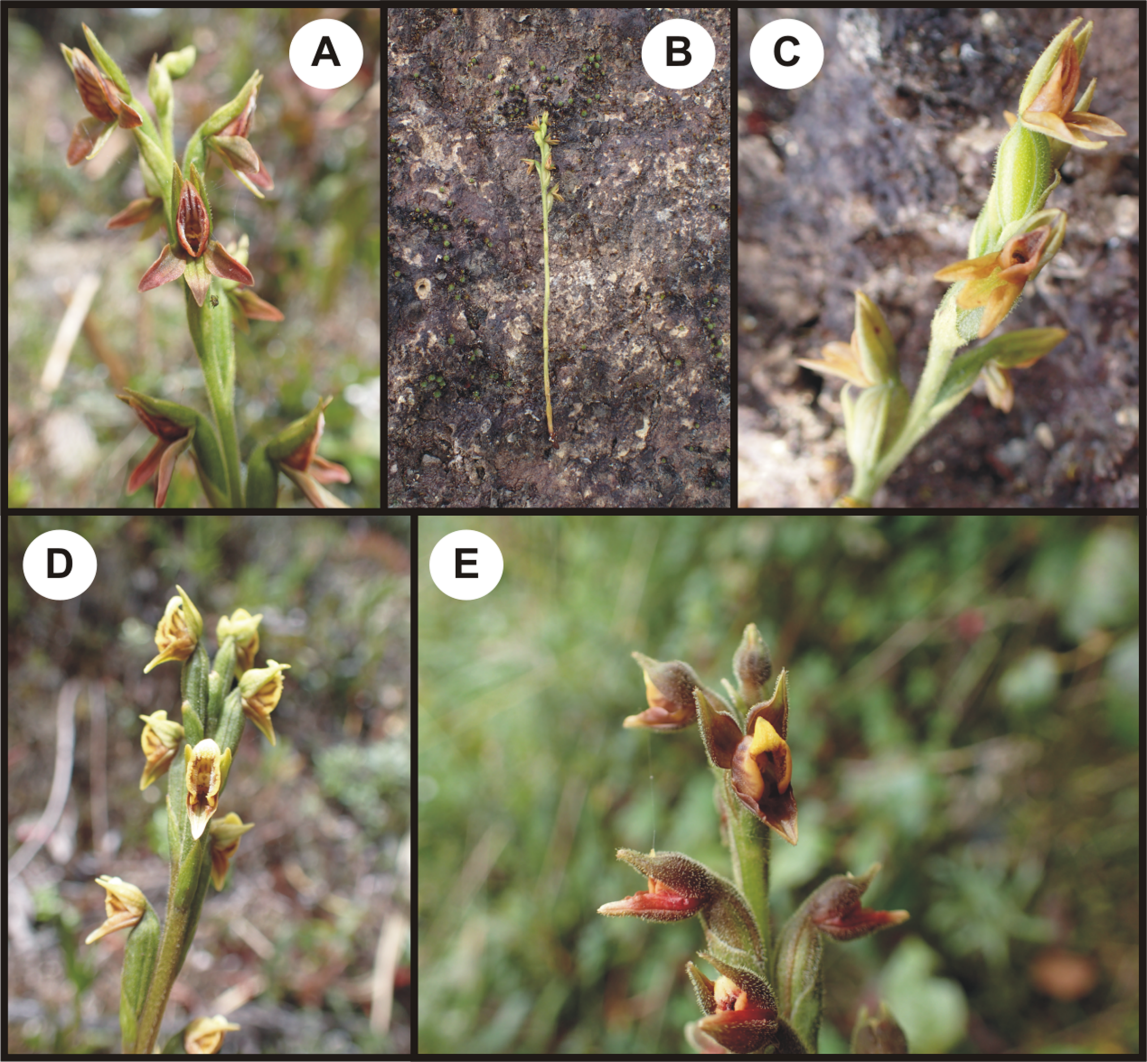
Ecuadorian *Pterichis* sect. *Pterichis* (A–C) and sect. *Acraea* (D and E). (A–C) *P. acuminata*. (D) *P. multiflora*. (E) *P. triloba*. Photos: M. Kolanowska.

**Figure 5:**
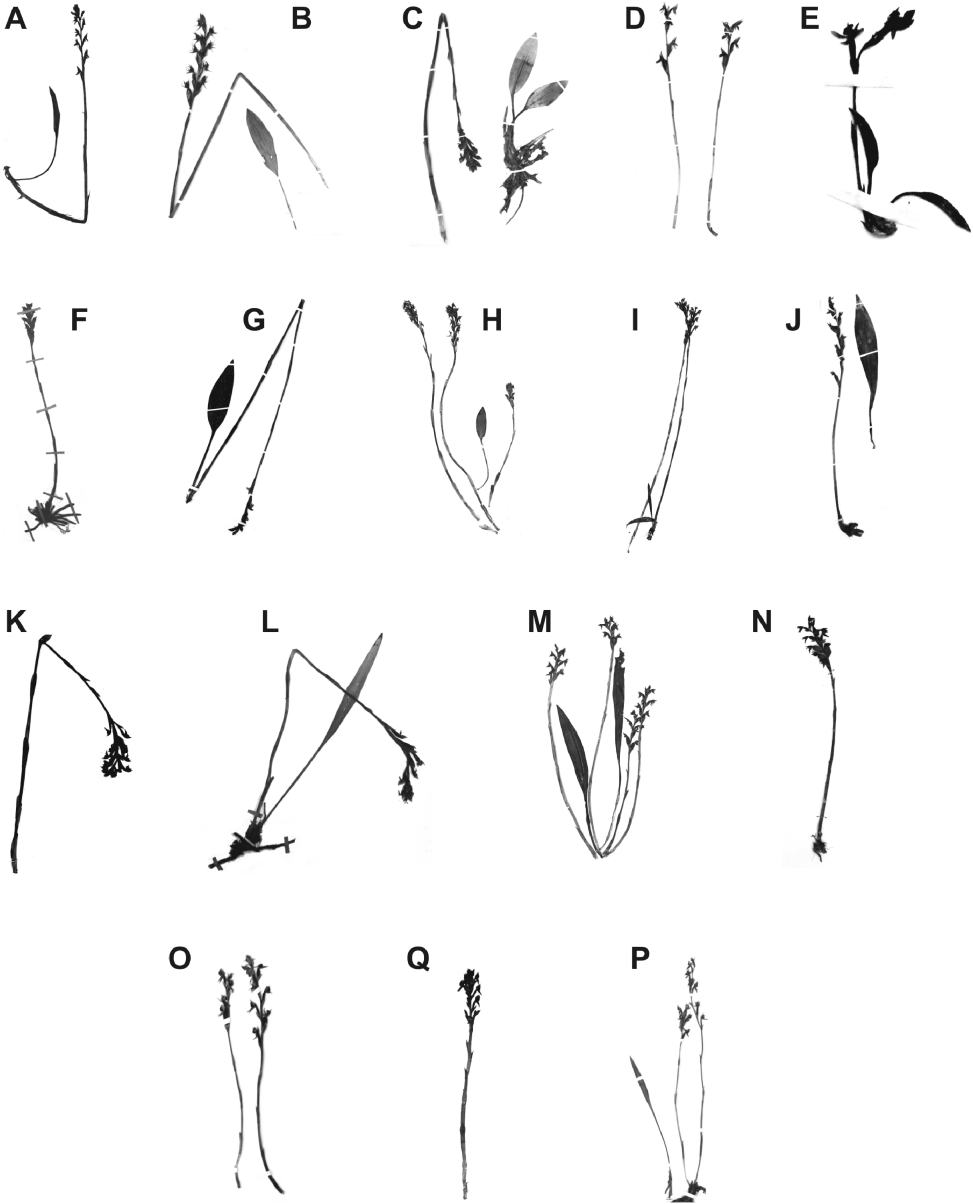
Habit of Ecuadorian *Pterichis* sect. *Pterichis* (A–G) and sect. *Acraea* (H–P). (A) *Pterichis acuminata—Dodson & Chase 17200* (RPSC). (B) *Pterichis aragogiana*—*Holm-Nielsen et al. 29519 (AAU)*. (C) *Pterichis diuris—Funck & Schlim 1218 (W)*. (D) *Pterichis galeata—Ulloa et al. 92355 (AAU)*. (E) *Pterichis meirax—Jameson s.n. (W)*. (F) *Pterichis ansaloniana—Ulloa et al. 1361 (HA)*. (G) *Pterichis madsenii—Madsen & Ellemann 75293 (AAU)*. (H) *Pterichis elliptica—528*. (I) *Pterichis habenarioides—Huttel 528 (QCNE)*. (J) *Pterichis multiflora—Løjtnant & Molau 13910 (AAU)*. (K) *Pterichis parvifolia—Álvarez et al. 2613 (QCNE)*. (L) *Pterichis pauciflora—Minga & Verdugo 2530 (HA)*. (M) *Pterichis seleniglossa—Dodson et al. 16396 (QCNE)*. (N) *Pterichis triloba—Dodson et al. 10837 (Q)*. (O) *Pterichis tunguraguona—Holm-Nielsen et al. 5108 (AAU)*. (Q) *Pterichis hirtziana—van der Werff & Palacios 8956 (QCNE)*. (P) *Pterichis dodsoniana—Holm-Nielsen et al. 4767 (AAU)*.

Terrestrial, rarely epiphytic, cespitose plants. Roots tuberous, fleshy, clustered, usually 1.5–5.0 mm in diameter. Leaves basal, few, rosulate, erect, shortly petiolate, blade usually narrow, lanceolate. Stem simple, loosely sheathed. Inflorescence loosely to subdensely few-flowered, lateral. Flowers non-resupinate, medium-sized, with the lateral sepals and lip uppermost, usually reddish, maroon, brownish, yellowish, or polychromatic, sometimes with dark freckles or lines. Ovary pubescent-glandular. Sepals subequal, free. Petals narrower than the dorsal sepal, free or adnate to the dorsal sepal. Lip sessile, very broad, concave in the center, variously ornamented with papillae or knob-like appendages along margins, more or less 3-lobed; lateral lobes broad, surrounding the gynostemium; middle lobe relatively narrow, fleshy, recurved. Gynostemium short, erect, massive. Column part rudimentary, much shorter than the anther, column foot missing. Anther erect, ovate or oblong-cordate, blunt to acute, motile. Pollinia 4, oblong-ovoid, relatively compact, soft and powdery in *P. triloba*. Caudiculae formed from the apical and elongate parts of pollinia. Staminodes thin and membranous or thick, fleshy, wing-like to lobed, joined with the filament and the style below the stigma base, or entirely free, enveloping the anther. Stigma ventral, confluent, flat to slightly convex, or horizontal, deeply concave, pocket-like in *P. triloba*. Rostellum erect, narrow, filiform, delicate, soft, or canaliculate with relatively broad base, truncate at the apex after the removal of the pollinium. Viscidium single, detachable, cellular, thick, small, rounded or hood-like, asymmetric. Hamulus usually present, finger-like, short or prominent, very obscure. Figs. 4 and 5.

The genus embraces about 45 species distributed from Costa Rica and Jamaica to Bolivia. The majority of them are known from the Andes.

### Key to the sections

1. Petals free from the dorsal sepalsect. *Pterichis*
1* Petals adnate to the dorsal sepalsect. *Acraea*

***Pterichis* section *Pterichis***

Petals free from the dorsal sepal.

### Key to section section *Pterichis*

1. Petals almost linear, about 10 times longer than wide, sepals basally ovate, with margins rolled up forming acuminate-caudate projection*P. aragogiana*
1* Petals ovate, elliptic to oblong-lanceolate, up to 5 times longer than wide, sepals apices obtuse to acuminate, but not as described above2
2. Floral bracts glabrous3
2* Floral bracts glandular or ciliate4
3. Petals glabrous, lip middle lobe constituting ca. 1/4 of the lip length*P. diuris*
3* Petals ciliate, lip middle lobe almost as long as the lip lamina*P. ansaloniana*
4. Lip basal part without auricles or with inconspicuous auricles5
4* Lip basal part with prominent auricles reaching almost the lip base*P. galeata*
5. Lip basal part reniform … 6
5* Lip basal part transversely elliptic; ***P. meirax***
6. Petals subequal in length to dorsal sepal, lip wider than long*P. acuminata*
6* Petals longer than dorsal sepal, lip equally long and wide*P*. *madsenii*

***Pterichis acuminata*** Schltr., Repert. Spec. Nov. Regni Veg. Beih. 7: 56. 1920. TYPE (Garay, 1978: 182): Colombia. Antioquia. Alt. 3,200 m. *M. Madero 27* (B†; Lectotype: AMES-00103701!—drawing).

**Figure 6:**
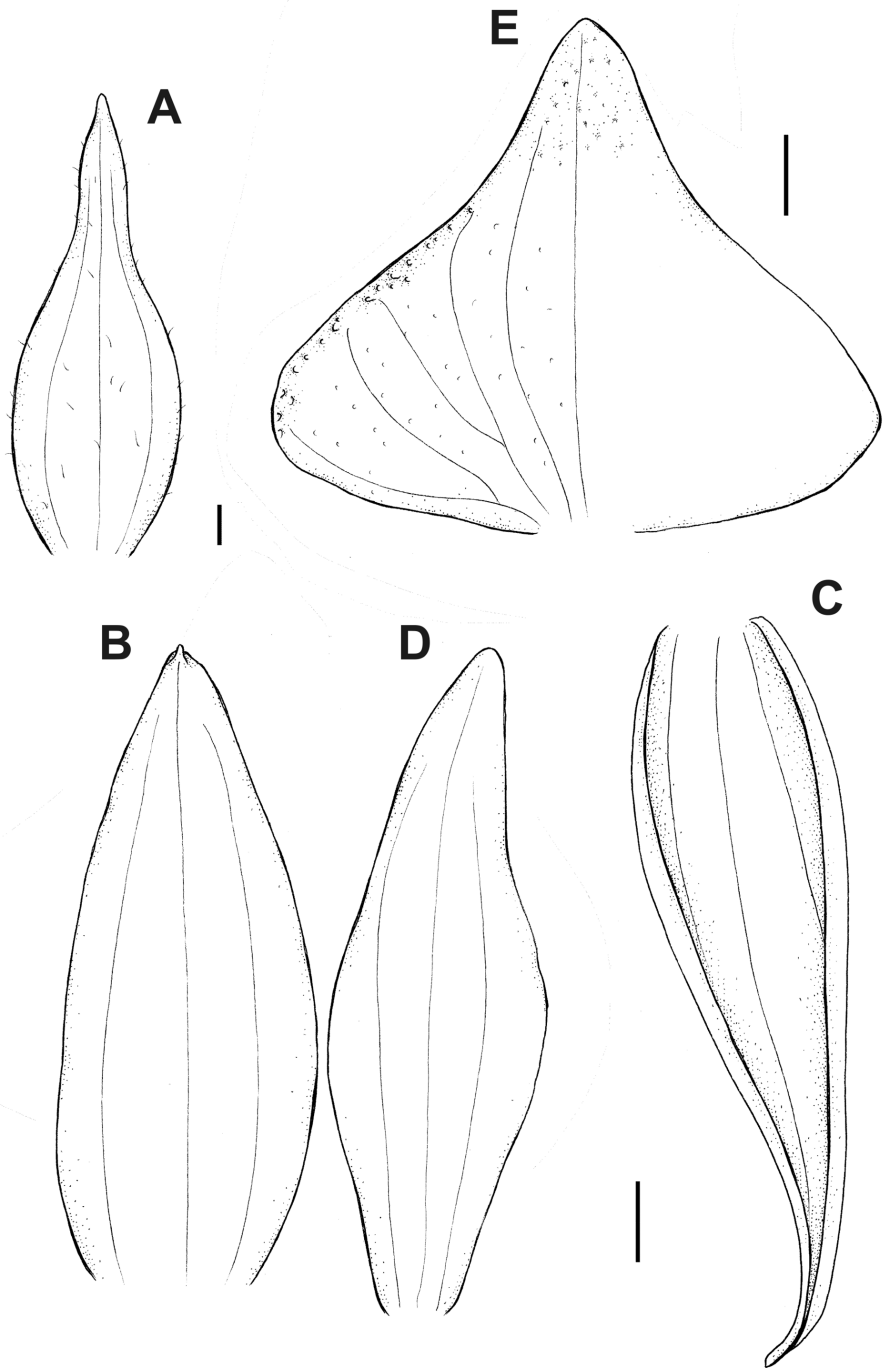
*Pterichis acuminata* Schltr. (A) Floral bract. (B) Dorsal sepal. (C) Lateral sepal. (D) Petal. (E) Lip. Scale bars = 1 mm. Drawn by S. Nowak from *Suin & Guartán 1014* (QCNE).

Plants 23–62 cm tall. Leaf, when present, petiolate; petiole 3.0–10.0 cm long; blade 5.5–10.0 × 2.5–4.0 cm, elliptic, acute. Scape glandular-ciliate in the upper part, glabrous at base, enclosed by 4–5 tubular sheaths. Inflorescence 4.0–22.0 cm long, 5–20-flowered, rachis densely ciliate. Flowers with pale green and faintly purplish tinted sepals which are yellowish tinted with brown inside, dirty pale brownish purple petals, and reddish-purple lip with darker brownish purple veins and white hairs. Floral bracts 8.0–12.3 mm long, lanceolate to ovate-lanceolate, acute, externally glandular-ciliate. Pedicellate ovary 8.5–14.0 mm long, densely glandular. Dorsal sepal 7.9–9.8 × 2.5–4.2 mm, oblong-ovate to ovate-ligulate, subacute, 3-veined, externally densely ciliate. Petals 7.5–10.0 × 1.8–4.0 mm, free from dorsal sepal, obliquely oblong-lanceolate to lanceolate-sagittate, subobtuse, 3-veined, glabrous. Lateral sepals 8.8–10.0 × 2.2–3.6 mm, oblong-ovate to elliptic-lanceolate, subacute to subobtuse, 3-veined, externally densely ciliate. Lip 5.6–7.4 × 7.5–8.5 mm, obscurely 3-lobed, basal part reniform above truncate base; middle lobe narrowly triangular to ovate, subobtuse, somewhat fleshy, recurved; disc papillate, with irregular, knob-like projections along margins. Gynostemium 2.7–4.0 mm long. Figs. 5 and 6.

*Habitat and ecology:* Terrestrial plants growing on roadsides, in cloud forest, humid montane forest and paramo at the altitudes of 2,300–3,870 m. Flowering occurs in January, April, June, and July.

*Notes:* This species is considered by some authors (Schweinfurth, 1941, 1958) as conspecific with *Pterichis galeata*, which is easily distinguished from *P. acuminata* by long-clawed petals being longer than dorsal sepal (by 10–20%) and auriculate lip basal part. In somewhat similar *P. meirax* the petals are oblong-lanceolate, with some hairs on the margin and the lip basal part is transversely elliptic.

*Representative specimens:* Ecuador. Prov. Azuay. Parque Nacional Cajas. Km 33 Cuenca-Molleturo. Sendero alrededor de Laguna Cucheros. Alt. 3,820–3,870 m. 17 January 2003. *C. Ulloa et al. 1260* (HA!). Prov. Morona-Santiago. Cantón Gualaquiza. Area de Bosque Vegetación Protectora Tambillo. Alt. 2,900 m. 26 June 2001. *L. Suin & J. Guartán 1014* (HA!, QCNE!). Prov. Morona-Santiago. Between San José de Raranga and San Miguel de Cuyes. Alt. ca 2900 m. 9 July 2017. *M. Kolanowska et al. E17/73* (photo!). Prov. Napo. Road Quito to Baeza, above Papallacta. On lava flow at Lago Papallacta. Alt. 3,100 m. 22 Jun 1987. *C.H. Dodson & M. Chase 17200* (RPSC!); Summit and uppermost N slopes of Cerro Sumaco. Virgin moist páramo. Alt. 3,700–3,840 m. 26 April 1979. *B. Løjtnant & U. Molau 12786* (AAU!); km 30 on road from El Carmelo towards La Bonita, SE of Santa Bárbara. Alt. ca. 2,600 m. 13 April 1979. *B. Løjtnant et al. 12402* (AAU!); E-facing ridge on the N side of Cerro Sumaco. Virgin moist páramo with scattered shrubs, on steep slope. Alt. 3,400–3,600 m. 25 April 1979. *B. Løjtnant & U. Molau 12744* (AAU!). Prov. Sucumbíos. El Mirador, Playon de San Francisco-Julio Andrade, km 12. Alt. 3,200–3,400 m. 11 Jul 1991. *C.H. Dodson, N. Williams & M. Whitten 18781* (RPSC!, UGDA-DLSz!—drawing).

Pterichis cf. acuminata

In the following collections the general plant and flower appearance correspond to *P. acuminata*, but some details of the examined specimens distinguish them from the typical form of this species:

– Prov. Imbabura. Cotacachi Cantón. Parroquia Plaza Gutierrez. Table Chupa, arriba de Apuela. Bosque húmedo montano. Alt. 2,300–3,000 m. 12 May 1992. *G. Tipaz et al. 1000* (QCNE!)—cf. *P. acuminata* but with prominent, truncate lip basal part
– Prov. Morona-Santiago. Road Gualaceo-El Limon. From the pass towards El Limon. Paramo or scrub just below paramo. Alt. 3,200–3,400 m. 8 February 1989. *H. van der Werff & W. Palacios 10516* (QCNE!)—cf. *P. acuminata* but with prominent, truncate lip basal part
– Prov. Carchi [Sucumbíos]. Road Tulcán-El Carmelo, at km 10. Roadbank in patch of montane shrub in bunchgrass paramo. Alt. 3,350 m. 27 May 1980. *H. Balslev & F. Quintana 23834* (AAU!)—cf. *P. acuminata* but petal elliptic, 1-veined
– Prov. Sucumbíos. Alt. ca. 3,300 m. 6 July 2017. *M. Kolanowska E17/38* (photo!)—cf. *P. acuminata* but with petals microscopically, sparsely ciliate along margin
– Prov. Chimborazo. Next to the road Toncal de la Sierra. 8 July 2017. Alt. 3,200 m. *M. Kolanowska et al. E17/5*1(photo!)—cf. *P. acuminata* but with prominent, truncate lip basal part and petals microscopically, sparsely ciliate along margin.

***Pterichis aragogiana*** Szlach. & Kolan., Ann. Bot. Fenn. 56(1–3): 176–178. 2019. TYPE: Ecuador. Prov. Azuay. Páramo de Matanga, km 25 on road Sigsig–Gualaquiza (old muletrack), W of the pass. Alt. 3,150 m. 14 December 1980. *L.B. Holm-Nielsen et al. 29519* (holotype AAU!).

**Figure 7:**
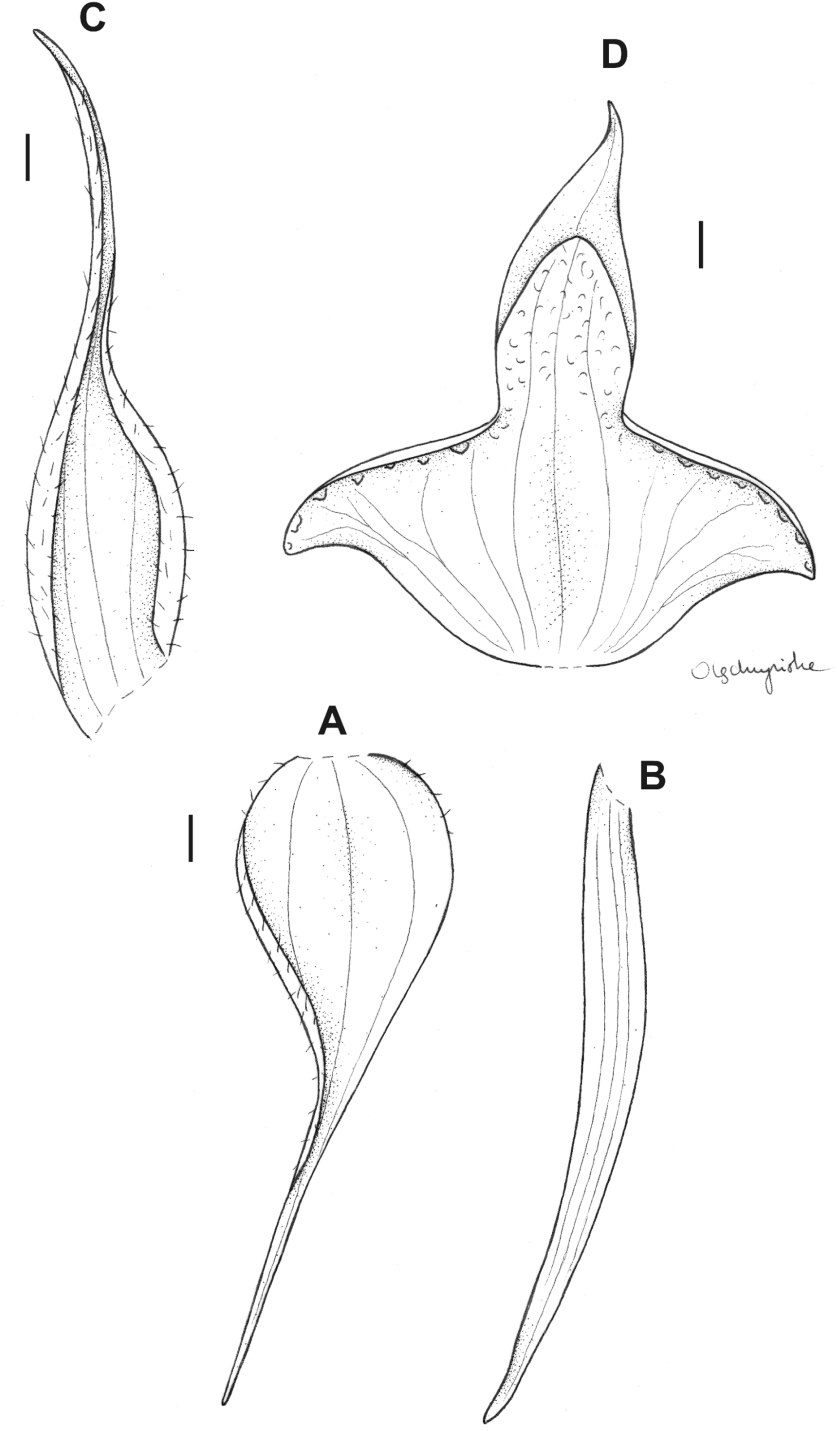
*Pterichis aragogiana* Szlach. & Kolan. (A) Dorsal sepal. (B) Lateral sepal. (C) Petal. (D) Lip. Scale bars = 1 mm. Drawn by N. Olędrzyńska from *Holm-Nielsen & al. 29519* (AAU).

Plants 40–55 cm tall, leafless during flowering. Scape glandular in upper third, enveloped in 7 tubular, acute sheaths of which upper ones are glandular-ciliate. Inflorescence ca. 8.5 cm long, densely ciliate. Flowers externally olive-green, petals and internal part of sepals orange-red, lip orange red flushed with violet in basal part of lip middle lobe, sepals externally densely glandular-ciliate. Floral bracts 14.0–20.0 mm long, ovate, cucullate, externally densely ciliate. Pedicellate ovary ca. 13.0 mm long, densely glandular-ciliate. Dorsal sepal 11.0–14.5 × 2.8–4.5 mm, ovate in lower half, apical part narrow, rolled up along margins hence appearing acuminate-caudate, subobtuse, 3-veined. Petals 11.0–14.5 × 1.0–1.4 mm, free from dorsal sepal, linear, subobtuse, 3-veined, glabrous. Lateral sepals 9.5–15 × 3–3.5 mm, obliquely ovate in lower half, rolled up along margins above giving an acuminate-caudate appearance, subobtuse, 3-veined. Lip 9.0–12.0 × 10.0–11.0 mm, hastate, basal part reniform above truncate base; middle lobe ca half of the lip length, ovate, acuminate; disc glandular, primarily 9-veined, lateral veins branching, ornamented with knob-like projections along margins. Gynostemium 3.0–5.8 mm long. Figs. 5 and 7.

*Habitat and ecology:* Terrestrial plants growing in scrub páramo with *Blechnum* L. (Blechnaceae), *Befaria* Mutis (Ericaceae), *Osteomeles* Lindl. (Rosaceae), and *Cortaderia* Stapf. (Poaceae) at the altitude of 3,150 m. Flowering in October and December.

*Notes: Pterichis aragogiana* is easily distinguished from all other genus representatives by the caudate sepals. Their lower part is more or less ovate and the upper one is rolled up along margins forming a caudate, acuminate projection. Unlike in other species of the nominal section of *Pterichis*, the lip middle lobe of *P. aragogiana* constitutes ca. half of the total lip length.

*Representative specimens:* Ecuador. Prov. Azuay. Páramo de Matanga, km 25 on road Sigsig–Gualaquiza (old muletrack), W of the pass. Alt. 3,150 m. 14 December 1980. *L.B. Holm-Nielsen et al. 29519* (AAU!). Prov. Morona-Santiago. Road from Sigsig to El Pangui. Páramo Matanga. 3°11′49″S, 78°46′08″W, 4 October 2003. *M. Blanco et al. 2515* (FLAS!). Prov. Zamora Chinchipe. Cerro Plateado. *Á.J. Pérez 1355* (QCA!).

***Pterichis diuris*** Rchb. f., Bonplandia (Hannover) 2: 10. 1854. TYPE: (Kolanowska & Olędrzyńska, 2015: 44): Venezuela. Mérida. *N. Funck & L.J. Schlim 1218* (lectotype W-R 377!, isolectotype AMES-00083567—microscope slide; AMES-00103705!—drawing)

**Figure 8:**
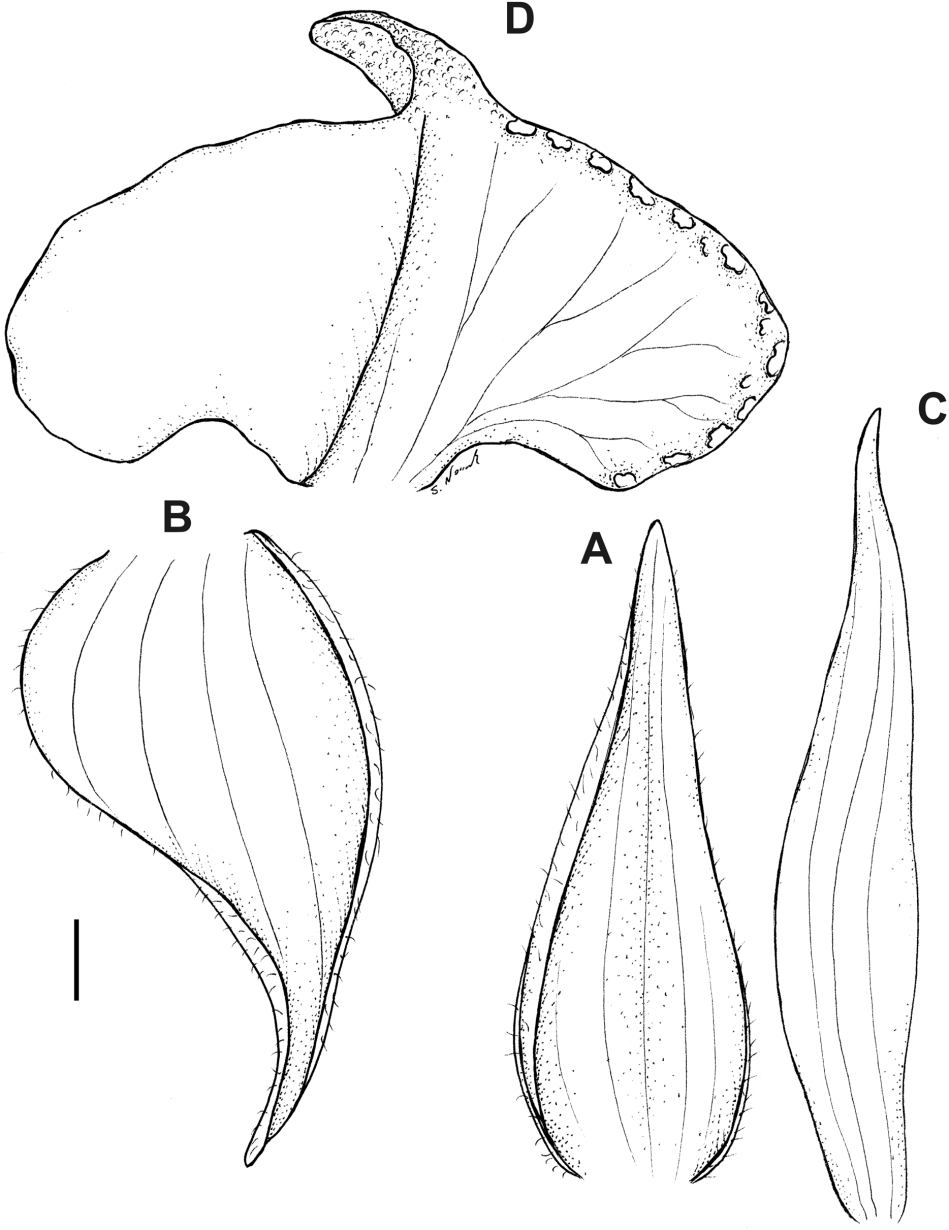
*Pterichis diuris* Rchb. f. (A) Dorsal sepal. (B) Lateral sepal. (C) Petal. (D) Lip. Scale bars = 1 mm. Drawn by S. Nowak from *Holm-Nielsen & al. 17385* (AAU).

Plants 40 cm long. Leaf up to 15.0 cm long, petiolate, petiole up to 7.0 cm long; blade 5.8–7.6 × 2.3 cm, oblong-elliptic, obtuse. Scape with 4–5 tubular, glabrous sheaths. Inflorescence 7.0–8.0 cm long, ciliate, subdensely several-flowered. Floral bracts 13.0 mm long, ovate, obtuse, glabrous. Pedicellate ovary 14.0 mm long, pubescent. Flowers with dirty green sepals, translucent, brownish-yellow petals and yellow, brown-veined lip. Dorsal sepal 8.3 × 3.0 mm, ovate-lanceolate, obtuse, 3- or 5-veined, externally ciliate. Petals 10.2 × 2.0 mm, free from dorsal sepal, obliquely linear-lanceolate, obtuse, 3-veined, glabrous. Lateral sepals 9.0 × 4.5 mm, obliquely ovate, subacute, 3- or 5-veined, externally sparsely ciliate. Lip 6.1 ×10.0 mm, basal part reniform in outline with lateral auricles, base truncate; middle lobe about 1/4 of the total lip length, narrowly triangular-lanceolate, papillate; disc ornamented with large glands along the margin, primarily 5-veined, lateral veins branching. Gynostemium 3.0 mm long. Figs. 5 and 8.

*Habitat and ecology:* Terrestrial plants growing in montane forest, in steep creek with large ferns at the altitude of 3,100–3,150 m. Flowering occurs in April.

*Notes:* From all other Ecuadorian species of nominal section of *Pterichis, P. diuris* differs in having glabrous floral bracts. In the presence of the auricles, the lip of this taxon resembles somewhat *P. galeata* which differs from *P. diuris* by obliquely elliptic petals which are not long-clawed.

*Representative specimen:* Ecuador. Prov. Napo. N side of Cerro Sumaco, loma NW of campsite. Alt. 3,100–3,150 m. 28 April 1979. *L.B. Holm-Nielsen et al. 17385* (AAU!).

***Pterichis galeata*** Lindl., Gen. Sp. Orchid. Pl. 445. 1840. TYPE (Kolanowska & Szlachetko, 2017: 91): Peru. *A. Mathews s.n*. (lectotype K-L-000079985!).

**Figure 9:**
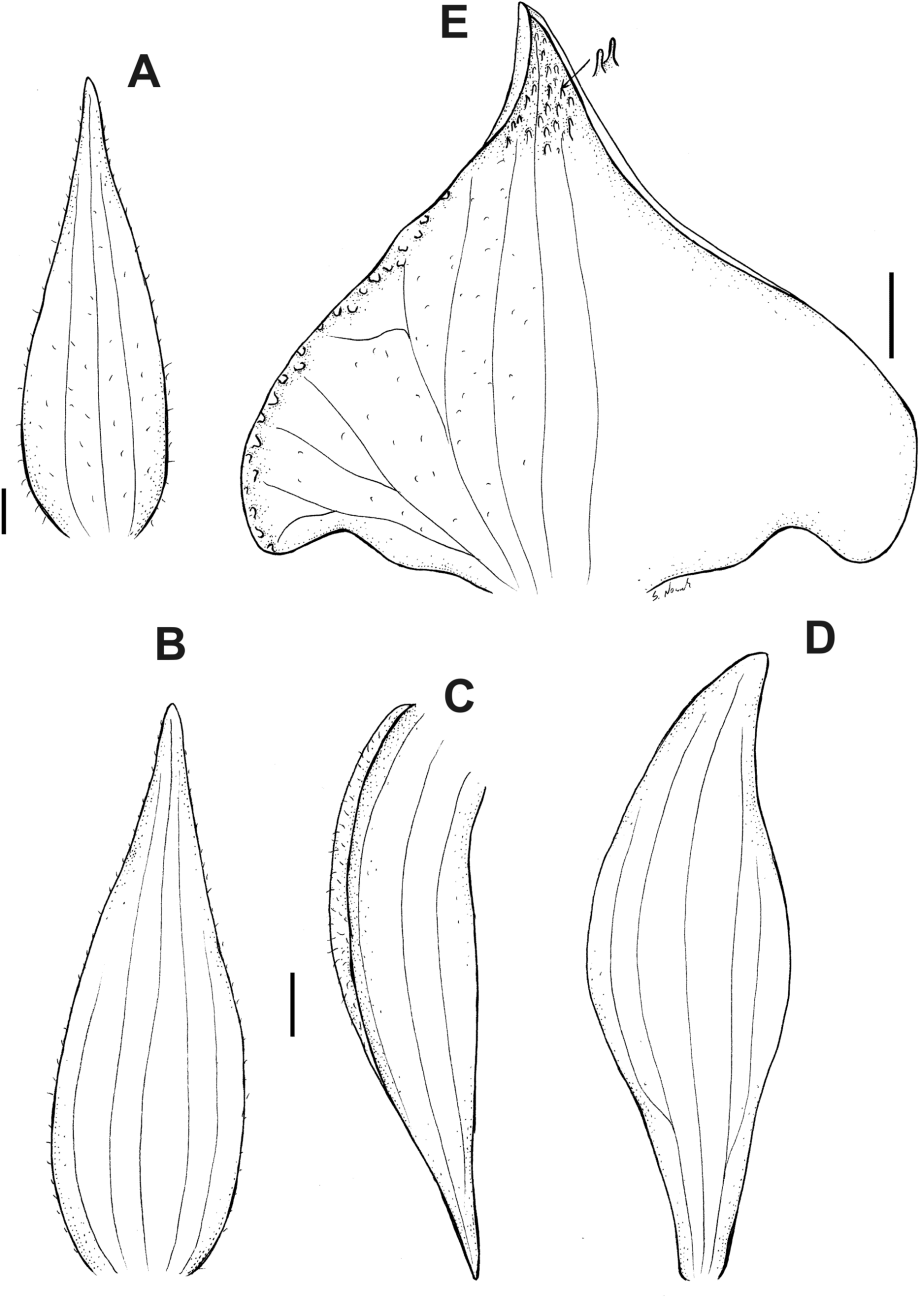
*Pterichis galeata* Lindl. (A) Floral bract. (B) Dorsal sepal. (C) Lateral sepal. (D) Petal. (E) Lip. Scale bars = 1 mm. Drawn by S. Nowak from *Ulloa & al. 92355* (AAU).

Plant 25–30 cm tall. Leaf (when present) petiolate; petiole 2.0 cm long; blade 4.0 × 1.0 cm, oblong-elliptic, obtuse. Scape glandular-pilose above, provided with 4–5, more or less distant, tubular sheaths, the lower 2 glabrous, the upper ones pilose. Inflorescence 4.0–8.0 cm long, sublaxly 5–7-flowered, rachis densely ciliate. Flowers greenish yellow with brown veins. Floral bracts ca. 11 mm, ovate to lanceolate, ciliate. Pedicellate ovary 13.0 mm long, densely ciliate. Dorsal sepal 9.2 × 3.0 mm, ovate-lanceolate, obtuse, 3-veined, externally ciliate. Lateral sepals 9.3 × 2.6 mm, obliquely lanceolate to lanceolate-ovate, acuminate, 3-veined, externally ciliate. Petals 10.0 × 3.3 mm, free from dorsal sepal, obliquely lanceolate-elliptic, unguiculate or narrowed below, obtuse, 3-veined, lateral veins branching, glabrous. Lip 7.1 × 8.3 mm, obscurely 3-lobed, basal part broadly reniform- with pair of auricles on edges; middle lobe triangular or triangular-lanceolate, densely hairy; disc papillate with series of irregular glands along margin. Gynostemium 3.0 mm long. Figs. 5 and 9.

*Habitat and ecology:* Terrestrial on roadside and in secondary vegetation dominated by *Freziera* Willd. (Theaceae) at the altitude of 3,050–3,100 m. Flowering in Ecuador occurs in August.

*Notes:* This species can be distinguished from other Ecuadorian representatives of *Pterichis* sect. *Pterichis* by having long-unguiculate petals. In examined Ecuadorian specimens the petal claw is shorter than in typical form of *P. galeata* but without additional material we are not able to define them as a new species.

*Representative specimen:* Ecuador. Prov. Carchi. Julio Andrade-El Carmelo, turn off towards El Ajún, km 0–3. Alt. 3,050–3,100 m. 10 August 1990. *C. Ulloa et al. 92355* (AAU!).

***Pterichis meirax*** Rchb. f. *ex* Szlach. & Kolan., Ann. Bot. Fenn. 51(5): 331. 2014. TYPE: Ecuador. *W. Jameson s.n*. (holotype W 870!).

**Figure 10:**
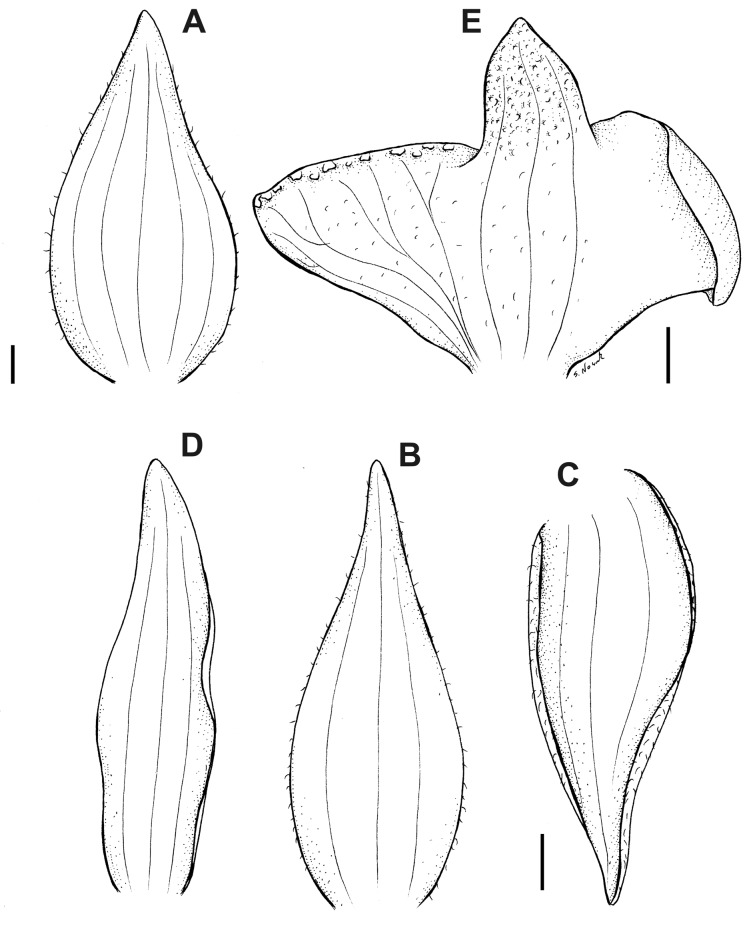
*Pterichis meirax* Rchb. f. *ex* Szlach. & Kolan. (A) Floral bract. (B) Dorsal sepal. (C) Lateral sepal. (D) Petal. (E) Lip. Scale bars = 1 mm. Drawn by S. Nowak from *Løjtnant & al. 13755* (AAU).

Plants 10–22 cm tall. Leaf petiolate; petiole 2.0–8.0 cm long; blade 3.5–13.0 × 0.9–1.4 cm, lanceolate to oblong-lanceolate, acute. Scape ciliate above the basal third, with 1 glabrous and 4 ciliate sheaths. Inflorescence 3.5–7.0 cm long, laxly 5–9-flowered, rachis densely ciliate. Flowers with dirty brownish-green sepals, dirty brownish purple petals tipped with yellow, and reddish to reddish-brown lip tipped with yellow and with white hairs. Floral bracts 6–17 mm long, externally ciliate. Pedicellate ovary 7–20 mm long, densely ciliate. Dorsal sepal 6.0–8.3 × 2.0–3.3 mm, ovate-lanceolate, obtuse, 3-veined, externally glandular to ciliate. Petals 7.0–8.0 × 2.3–3.0 mm, free from dorsal sepal, obliquely oblong-lanceolate to linear-lanceolate, subobtuse, glabrous, 3-nerved. Lateral sepals 6–8.2 × 2.5–3.2 mm, obliquely ovate, concave, acute, 3-veined, externally glandular to ciliate. Lip 5.5–6.3 × 8.0–9.0 mm, basal part transversely elliptic in outline above truncate base; middle lobe ca 1/3 of the lip length, ovate, papillate; disc papillate with numerous knob-like projections along margin in the upper half, 7- or 9-veins with lateral veins branching. Gynostemium 3.0–3.6 mm long. Figs. 5 and 10.

*Habitat and ecology:* Terrestrial in elfin forest and steep slopes along road at the altitude of 3,200–3,500 m. Flowering occurs in April and May.

*Notes:* This species resembles *Pterichis acuminata* from which it differs by the petals shape (oblong-lanceolate in *P. meirax*) and transversely elliptic lip basal part (reniform in *P. acuminata*). *P. meirax* resembles *P. habenarioides* from which it differs mainly by its lip form. The lip of *P. habenarioides* is triangular-elliptic in outline, with subtriangular apical part, pubescent margins and interior papillae. The papillae on *P. meirax* lip are distributed also along the thickened apical margins below the ligulate apex.

*Representative specimens:* Ecuador. Prov. Carchi. Carmelo, road from Tulcán to Santa Barbara. Alt. 3,200 m. May 1985. *A. Hirtz 2617* (RPSC!, UGDA-DLSz!—drawing). Prov. Cotopaxi. Latacunga-Quevedo road, above Pilaló, km 74 from Pujili. Alt. 3,500 m. 26 April 1979. *B. Løjtnant et al. 13755* (AAU!). Prov. Tungurahua. *W. Jameson s.n*. (W!).

***Pterichis ansaloniana*** Kolan., Szlach. & S. Nowak, ***sp. nov*.** TYPE: Ecuador. Prov. Azuay. Parque Nacional Cajas. Km 35.7 Cuenca-Molleturo. Sendero Tres Cruces. Laguna Negra-Laguna Larga. Páramo de pajonal. Alt. 4,100 m. 23 January 2003. *C. Ulloa et al. 1361* (holotype HA 5333!—left hand plant; isotype HA 5333!).

*Species similar to* Pterichis diuris *distinguished by more or less lanceolate, ciliate petals, broadly truncate lip base, presence of prominent knob-like projections on the lip margins, and larger lip middle lobe*.

**Figure 11:**
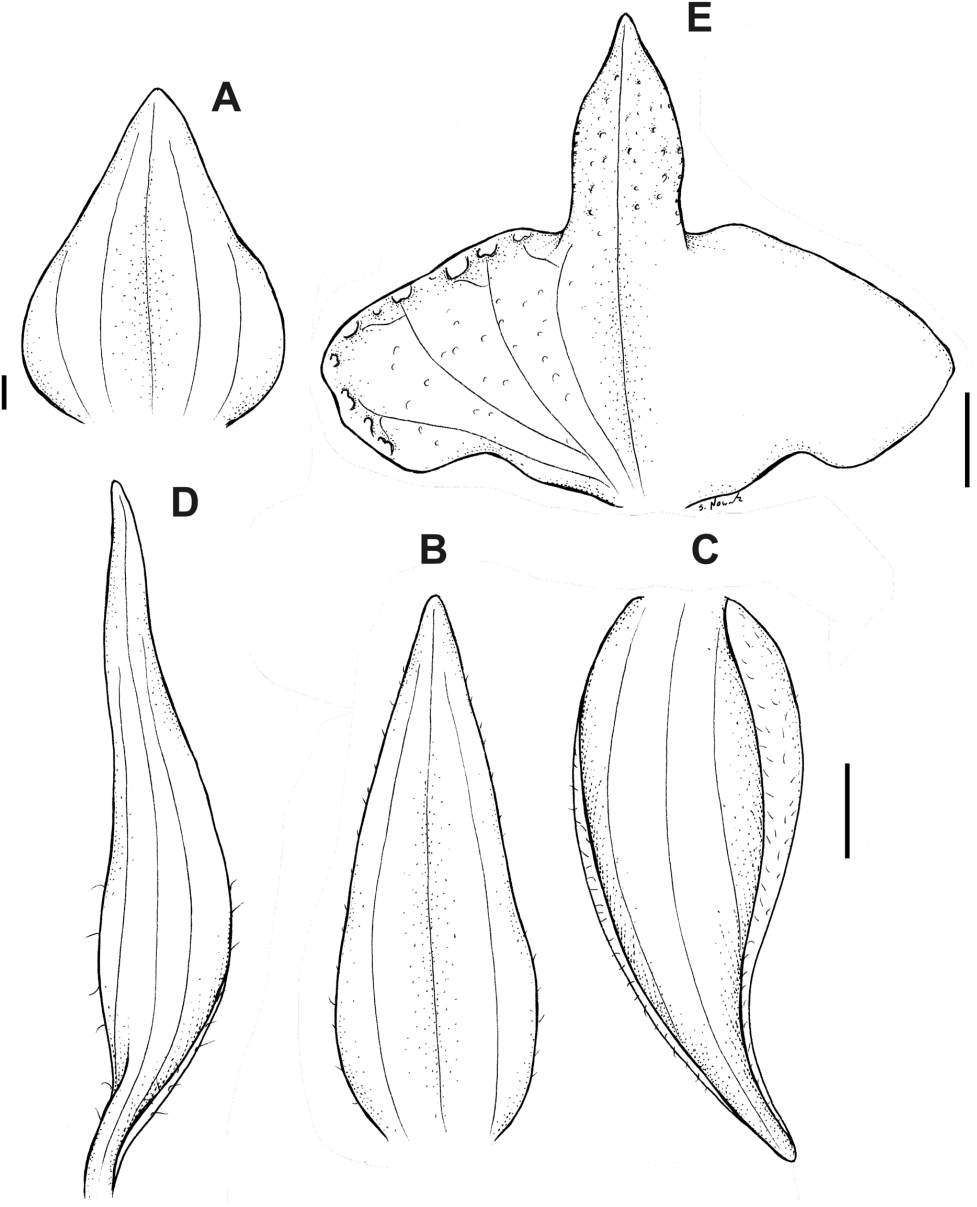
*Pterichis ansaloniana* Kolan., Szlach. & S. Nowak, *sp. nov*. (A) Floral bract. (B) Dorsal sepal. (C) Lateral sepal. (D) Petal. (E) Lip. Scale bars = 1 mm. Drawn by S. Nowak from *Ulloa & al. 1361* (HA).

Plant 18–30 cm tall. Leaf unknown. Scape with 5–6 prominent sheaths. Inflorescence ca. 6 cm long, congested, few-flowered, rachis ciliate. Floral bracts 10 mm long, ovate, rounded, glabrous. Pedicellate ovary 13.5 mm long, sparsely ciliate. Flowers with green sepals and yellow lip with brown-purple lines. Dorsal sepal 6.0 × 2.4 mm, oblong ovate, rounded at the apex, 3-veined, externally sparsely ciliate. Petals 8.9 × 1.8 mm, free from dorsal sepal, obliquely lanceolate, widened in the basal third, 3-veined, sparsely ciliate. Lateral sepals 6.5 × 2.7 mm, obliquely ovate, acuminate, obtuse, 3-veined, externally sparsely ciliate. Lip 5.5 × 7 mm, reniform above truncate base; middle lobe ca half of the lip length, elliptic, apiculate, papillate; disc papillate with series of knob-like projections along the margin. Gynostemium 2.8 mm long. Figs. 5 and 11.

*Etymology*: Dedicated to Raffaella Ansaloni, the curator of Herbario Azuay.

*Habitat and ecology:* Terrestrial in paramo at the altitude of 4,100 m. Flowering in January.

*Notes:* This species resembles *Pterichis diuris* from which it can be distinguished by more or less lanceolate, ciliate petals (vs. glabrous, obliquely elliptic), broadly truncate lip base, presence of prominent knob-like projections on the lip margins (vs. with large glands along the margin), and larger lip middle lobe (almost as long as the lip lamina vs. constituting ca. 1/6 of the lip length). The similar, long lip middle lobe is observed in *P. aragogiana*, but it this species sepals are caudate and petals are linear and glabrous.

Type collection deposited in HA consists of two plant, and the holotype is plant on the left hand with mature inflorescence.

*Representative specimen:* Ecuador. Prov. Azuay. Parque Nacional Cajas. km 35.7 Cuenca-Molleturo. Sendero Tres Cruces. Laguna Negra-Laguna Larga. Páramo de pajonal. Alt. 4,100 m. 23 January 2003. *C. Ulloa et al. 1361* (HA!).

***Pterichis madsenii*** Kolan., Szlach. & S. Nowak, ***sp. nov*.** TYPE: Ecuador. Prov. Loja. Parque Nacional Podocarpus. Above Nudo de Cajanuma, trail to Mirador. Scrub and ridge-top vegetation above tree limit. Alt. 3,000–3,150 m. 6 September 1988. *J.E. Madsen & L. Ellemann 75293* (holotype AAU!).

*Species similar to Pterichis acuminata but with sparsely ciliate sepals, prominent lip middle lobe and lip being as long as wide*.

**Figure 12:**
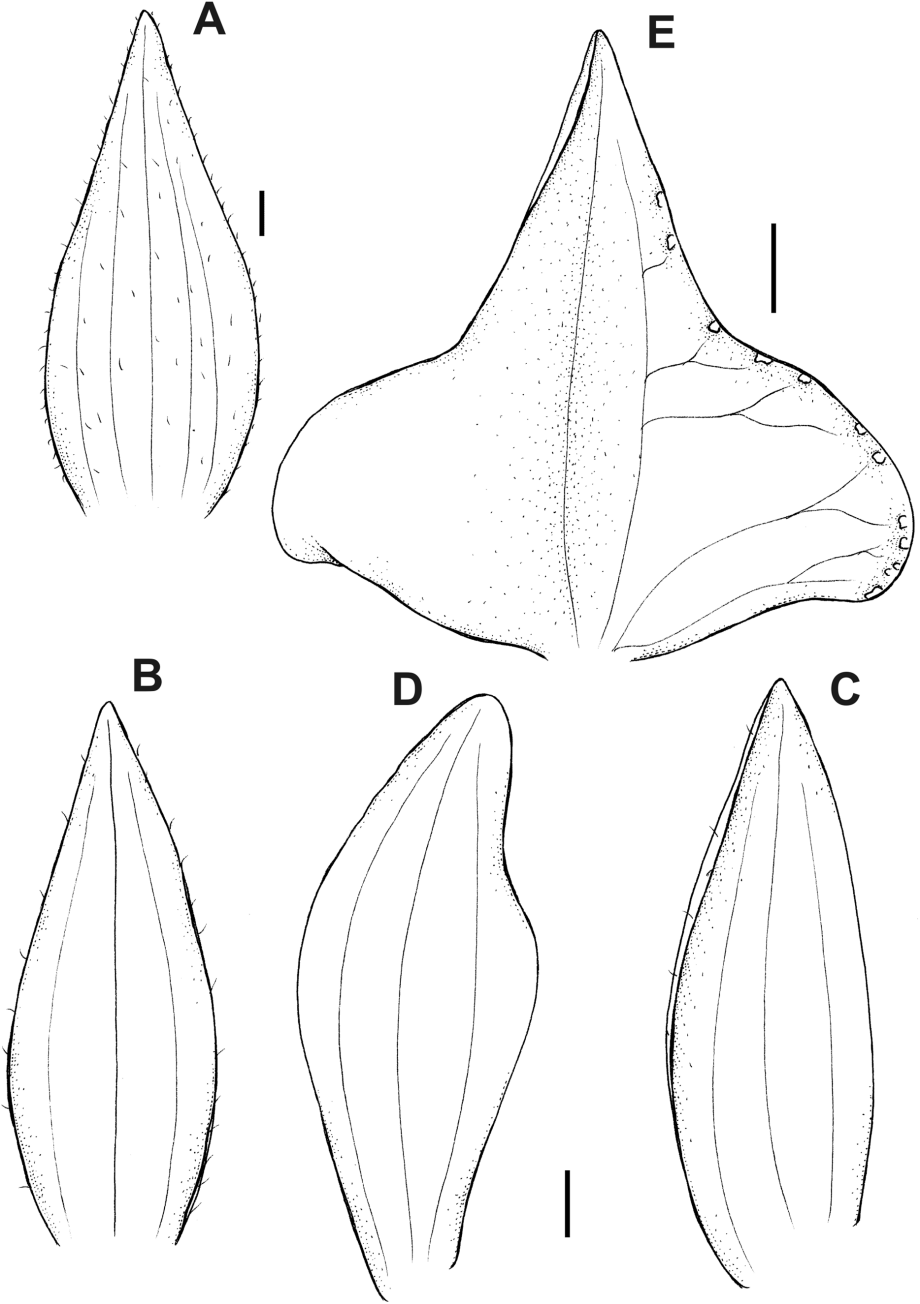
*Pterichis madsenii* Kolan., Szlach. & S. Nowak, *sp. nov*. (A) Floral bract. (B) Dorsal sepal. (C) Lateral sepal. (D) Petal. (E) Lip. Scale bars = 1 mm. Drawn by S. Nowak from *Madsen & Ellemann 75293* (AAU).

Plants ca. 70 cm tall. Leaf petiolate; petiole 12.0 cm long; blade 13.0 × 3.0 cm, narrowly elliptic, acute. Scape glandular-ciliate above basal part, enclosed by 8 tubular sheaths. Inflorescence 9.0 cm long, ca. 10-flowered, rachis densely ciliate. Flower color not recorded. Floral bracts ca. 11.0 mm long, ovate, acute, externally glandular-ciliate. Pedicellate ovary 14.0 mm long, pubescent. Dorsal sepal 8.0 × 3.0 mm, oblong-ovate, obtuse, 3-veined, externally sparsely ciliate. Petals 9.0 × 3.8 mm, free from dorsal sepal, obliquely elliptic, sub-bilobulate, rounded, 3-veined, glabrous. Lateral sepals 9.2 × 3 mm, oblong-ovate, subobtuse, 3-veined, externally sparsely ciliate. Lip 7.0 × 7.1 mm, basal part reniform, subauriculate; middle lobe ca half of the lip length, triangular, obtuse, fleshy; disc papillate, with irregular, knob-like projections along margins, 7-veined, veins apically branching. Gynostemium 3.0 mm long. Figs. 5 and 12.

*Etymology*: Dedicated to Jens E. Madsen, senior collector of the type specimen.

*Habitat and ecology:* Terrestrial in scrub and ridge-top vegetation above tree limit at the altitudes of 3,000–3,150 m. Flowering in September.

*Notes:* Species similar to *Pterichis acuminata* but with sparsely ciliate sepals (vs. sepals densely ciliate), prominent lip middle lobe constituting half of the total lip length (vs. up to 1/3 of the total lip length) and lip being as long as wide (vs. wider than long).

*Representative specimen:* Ecuador. Prov. Loja. Parque Nacional Podocarpus. Above Nudo de Cajanuma, trail to Mirador. Alt. 3,000–3,150 m. 6 September 1988. *J.E. Madsen & L. Ellemann 75293* (AAU!).

***Pterichis section Acraea*** (Lindl.) Kolan. & Szlach.

Ann. Missouri Bot. Gard. 102(1): 97. 2017. ≡ *Acraea* Lindl., Pl. Hartw.: 155. 1845; Type: *Acraea parvifolia* Lindl., Pl. Hartw.: 155. 1845.

Petals adnate to the dorsal sepal.

### Key to section Section *Acraea*

1. Petals glabrous2
1* Petals ciliate6
2. Truncate basal part of the lip constituting less than 1/3 of the lip lamina3
2* Truncate basal part of the lip constituting about 1/2 of the lip lamina5
3. Petals narrowly ovate above narrow claw*P. tunguraguona*
3* Petals linear-oblong to almost filiform4
4. Floral bracts densely glandular-pubescent*P. multiflora* var. *multiflora*
4.* Floral bracts glabrous*P. multiflora* var. *ecuadorensis*
5. Petals 1-veined*P. seleniglossa*
5* Petals 3-veined*P. triloba*
6. Lip basal part transversely elliptic, widest near the middle7
6* Lip basal part more or less reniform or lunate, widest below the middle8
7. Lip middle lobe broadly ovate-ligulate, constitutes less than 1/3 of the total lip length*P. elliptica* var. *elliptica*
7.* Lip middle lobe oblong-elliptic, constitutes more than 1/3 of the total lip length*P. elliptica* var. *ecuadorensis*
8. Lip lamina lunate*P. hirtziana*
8* Lip lamina reniform9
9. Lip triangular in outline with middle lobe not well-separated … ***P. pauciflora***
9* Lip with distinctly separated middle lobe 10
10. Lip lacking knob-like projections along the margin11
10* Lip with knob-like projections along the margin*P. parvifolia*
11. Petals 1.2 mm wide, linear, lip middle lobe constituting over 1/3 of the total lip length*P. dodsoniana*
11* Petals 1.5–2.5 mm wide, obliquely lanceolate, lip middle lobe constituting not more than 1/4 of total lip length*P. habenarioides*

***Pterichis elliptica*** Kolan. & Szlach., Wulfenia 22: 222. 2015. TYPE: Ecuador. Zamora-Chinchipe, Road from Loja to Zamora, km 14. Alt. 2,800 m. 18 November 1961. *C.H. Dodson & L.B. Thien 1326* (holotype RPSC 0000936-H!; isotype: RPSC 0000936-I!).

**Figure 13:**
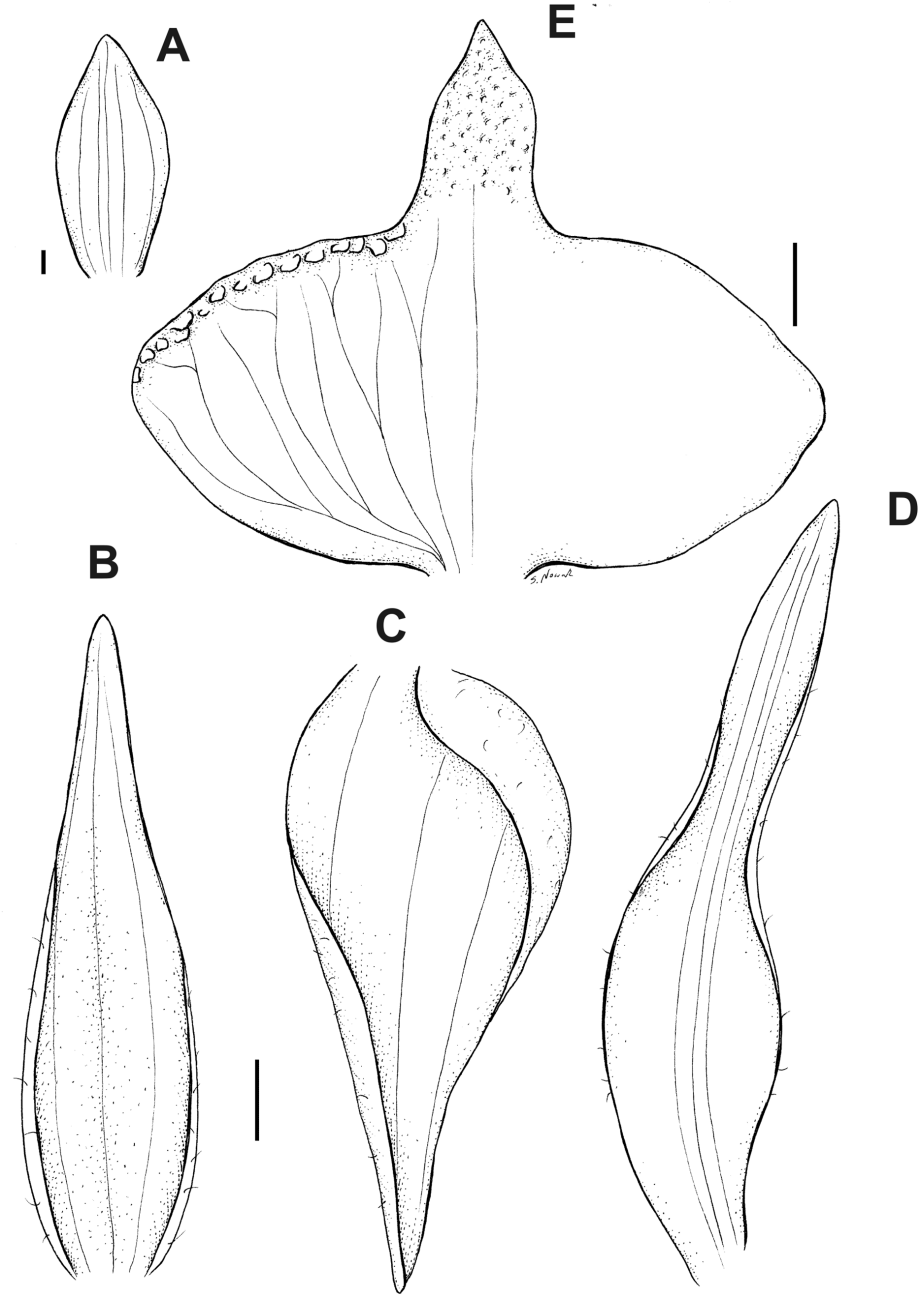
*Pterichis elliptica* Kolan. & Szlach. (A) Floral bract. (B) Dorsal sepal. (C) Lateral sepal. (D) Petal. (E) Lip. Scale bars = 1 mm. Drawn by S. Nowak from *Huttel 528* (QCNE).

Plants (12)27–40 cm tall. Leaf petiolate; petiole 5.0–9.0 cm long, blade up to 6.0 × 2.0 cm, elliptic, acute. Scape sparsely glandular at the base, densely near the apex, with 4–5 sheaths. Inflorescence 3.0-8.0 cm long, 3–9-flowered, rachis densely ciliate. Flowers greenish yellow or brownish yellow with maroon stripes. Floral bracts 7.2–11.8 mm long, elliptic-lanceolate to elliptic, obtuse to acuminate, glabrous or almost so. Pedicellate ovary 11.0–18.0 mm long, densely glandular-ciliate. Dorsal sepal 5.5–8.3 × 1.6–2.5 mm, ovate to ovate-lanceolate, obtuse, 3-veined, externally almost glabrous, glandular-ciliate near the base. Petals 5.2–10.0 × 1.5–2.2 mm, agglutinate to dorsal sepal, obliquely oblong-lanceolate, obtuse, 3-veined, sparsely ciliate. Lateral sepals 6–8 × 3–3.8 mm, obliquely ovate, obtuse, apically canaliculated, 3- or 4-veined, externally ciliate. Lip 5.3–7.0 × 7.5–8.8 mm, basal part transversely elliptic; middle lobe ca 1/3 of the lip length, broadly ovate-ligulate, papillate; disc papillate, with numerous knob-like projections along the margins, 9- or 11-veined. Gynostemium 2.3–3.2 mm long. Figs. 5 and 13.

*Habitat and ecology:* Terrestrial plant growing in trail embankment, montane forest and wet páramo vegetation with numerous shrubs or large patches of *Neurolepis* Meins. (Poaceae) or with *Blechnum* and thick layers of mosses at the altitude of 2,500–3,700 m. Flowering occurs in January, April, September, October, November and December

*Notes: Pterichis elliptica* resembles *P. parvifolia* from which it differs by the elliptic basal lip part (vs. reniform), rounded lip base (vs. truncate), and lip middle lobe constituting ca. 1/3 of the lip length (vs. middle lobe shorter than 1/2 of the total lip length).

*Representative specimens:* Ecuador. Prov. Azuay. Road Sigsig-Gualaquiza, km 25.6. At the pass on military post road. Wet páramo vegetation with large patches of *Neurolepis*. Km 3.3. from pass to military post. Alt. 3,200–3,330 m. 11 January 2000. *P.M. Jørgensen et al. 1810* (QCNE!). Prov. Loja. Along path (camino de herradura) at pass at km 13.5 Loja-Zamora. Alt. 2,800 m. 21 Sep 1980. *C.H. Dodson, C. Luer, J. Luer, P. Morgan, H. Morgan, A. Perry & J. Kuhn 10519* (RPSC!); Parque Nacional Podocarpus. Cerro Toledo. Montane forest and paramo. Alt. 2,500–3,400 m. 30 October 1989. *J.E. Madsen 86289* (AAU!, LOJA!, QCA!). Prov. Morona-Santiago. Sigsig-Gualaquiza, km 26, turnoff towards military antenas. Páramo with many shrubs. Alt. 3,290–3,570 m. 2 December 1990. *P.M. Jørgensen et al. 92777* (AAU!, QCA!). Prov. Napo. N side of Cerro Sumaco. 100 m NW of campsite. Alt. 3,700 m. 24 April 1979. *L.B. Holm-Nielsen et al. 17128* (AAU!). Prov. Pichincha. Papallacta. Alt. 3,600 m. 27 December 1984. *Ch. Huttel 528* (QCNE!). Prov. Zamora-Chinchipe. Road from Loja to Zamora, km 14. Alt. 2,800 m. 18 November 1961. *C.H. Dodson & L.B. Thien 1326* (RPSC 0000936-H!, RPSC 0000936-I!).

The below listed collection could be classified in *P. elliptica* but it is characterized by a very long lip middle lobe, not observed in other populations. Moreover, it was found growing epiphytically on *Polylepis* Ruiz & Pav. (Rosaceae) in humid montane forest while *Pterichis* almost always are terrestrial plants. In our opinion these features are sufficient to consider that as separated variety.

**Figure 14:**
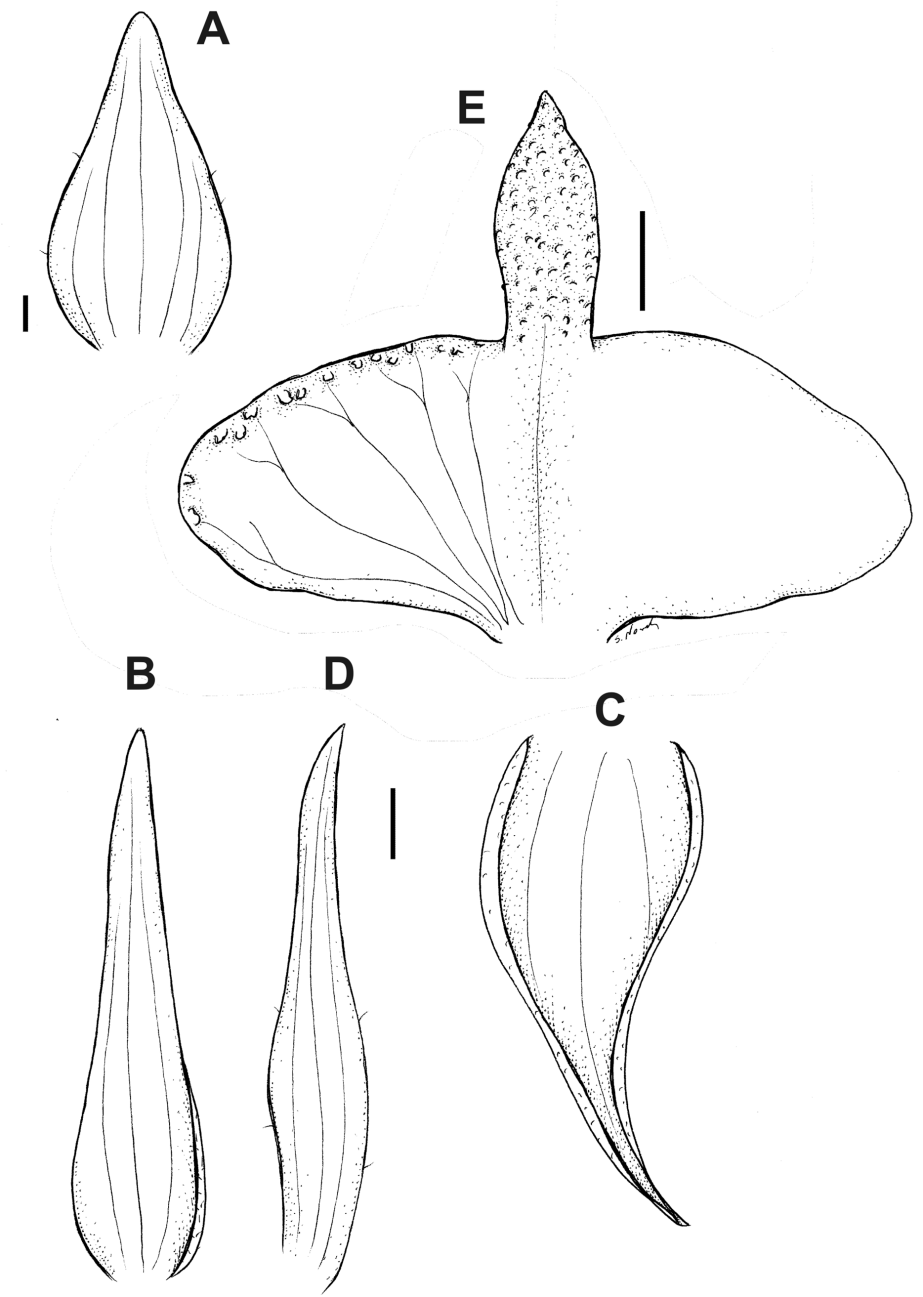
*Pterichis elliptica* var. *ecuadorensis* Kolan., Szlach. & S. Nowak, *var. nov*. (A) Floral bract. (B) Dorsal sepal. (C) Lateral sepal. (D) Petal. (E) Lip. Scale bars = 1 mm. Drawn by S. Nowak from *Teran & Quela 26* (QAP).

***Pterichis elliptica* var. *ecuadorensis*** Kolan., Szlach. & S. Nowak, ***var. nov*.** TYPE: Ecuador. Prov. Napo. Cantón Quijos. Parroquia Papallacta, sector Río Papallacta. Alt. 3,400 m. 2 November 1997. Flowers green. *S. Teran & P. Quela 26* (holotype: QAP 33285!). Fig. 14.

*Distinguished from typical form of* Pterichis elliptica *by the epiphytic habit, by the long, oblong-elliptic lip middle lobe which constitutes more than 1/3 of the total lip length*.

*Etymology*: In reference to the country of origin of the type collection.

***Pterichis habenarioides*** (F. Lehm. & Kraenzl.) Schltr., Repert. Spec. Nov. Regni Veg. Beih. 7: 214. 1920. ≡ *Goodyera habenarioides* F. Lehm. & Kraenzl., Bot. Jahrb. Syst. 26: 499. 1899. TYPE (Garay, 1978: 183): Colombia. Cauca. Paramo Guanacas. *F. Lehmann 6419* (lectotype K-000881685!).

*Pterichis costaricensis* Ames & C. Schweinf., Sched. Orch. 10: 10. 1930. TYPE (Garay, 1978: 183): Costa Rica. *H. E. Stork 2344* (lectotype: AMES-00103704!, isolectotypes: AMES-00083566!—microscope slide, US; UGDA-DLSz!—drawing).

**Figure 15:**
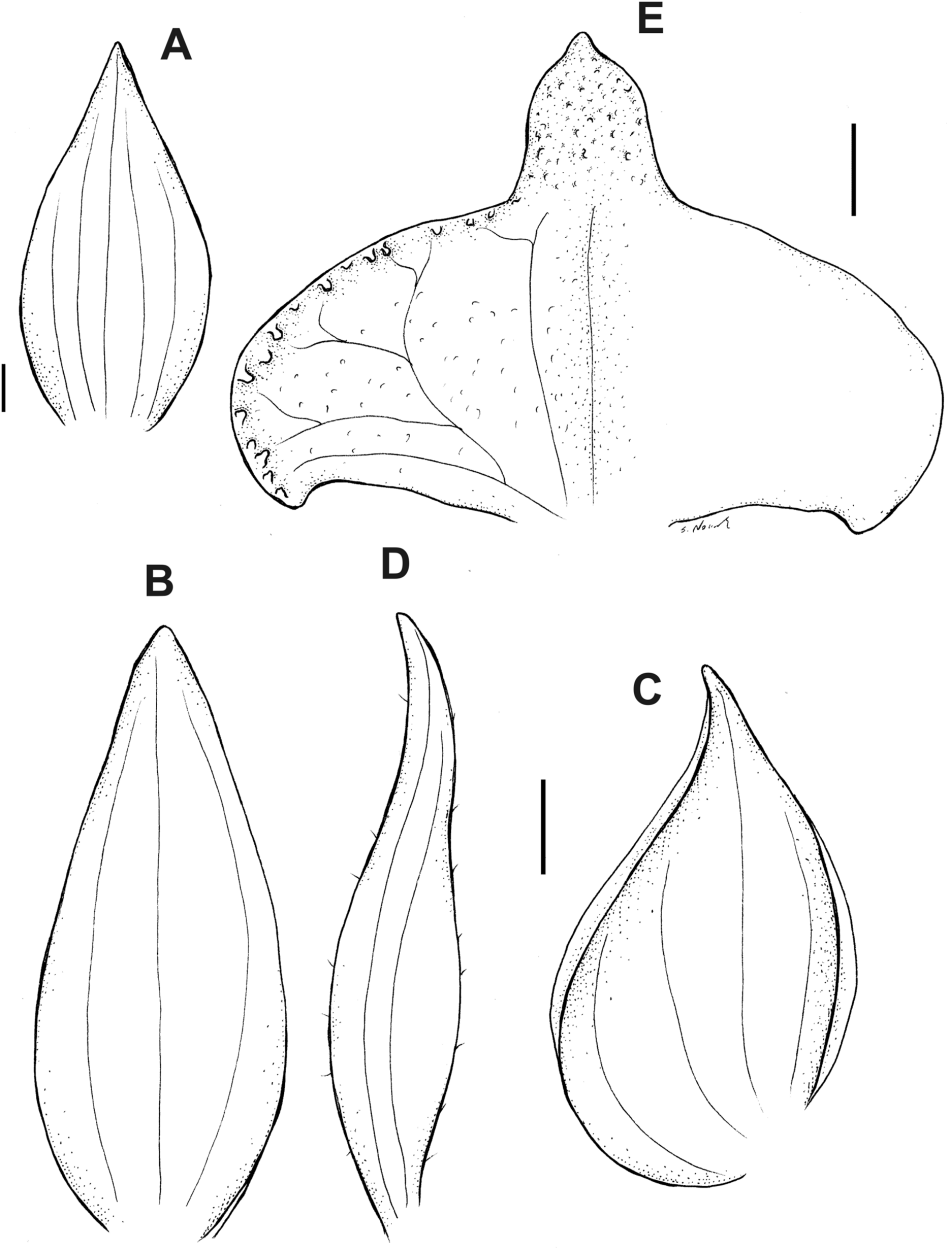
*Pterichis habenarioides* (F. Lehm. & Kraenzl.) Schltr. (A) Floral bract. (B) Dorsal sepal. (C) Lateral sepal. (D) Petal. (E) Lip. Scale bars = 1 mm. Drawn by S. Nowak from *Narváez 586* (QCNE).

Plants 34 cm tall. Leaf usually petiolate; petiole up to 12.0 cm long; blade 7.0–12.0 × 1.0–2.0 cm, narrowly lanceolate, acuminate. Scape pubescent, remotely 4–5-sheathed, sheaths glabrous. Inflorescence 7.0–10.0 cm long, sublaxly 10–12-flowered, rachis densely ciliate. Flowers yellow to green, sepals brown-spotted, petals greenish tinted, lip varying from deep green to yellow with dark purple or brownish markings. Floral bracts 8.3–9.0 mm long, ovate-lanceolate to ovate, obtuse, glabrous or only basally ciliate. Pedicellate ovary 9.0–11.5 mm long, puberulent. Dorsal sepal up 6.7–9.0 × 2.5–3.0 mm, ovate, obtuse, 3-veined, glabrous or externally sparsely ciliate. Petals 6.9–8.3 × 1.5–2.5 mm, agglutinate to dorsal sepal, obliquely lanceolate, falcate, with ligulate terminal third, 2- or 3-veined, sparsely ciliate along margins. Lateral sepals 5.7–7.8 × 3–3.8 mm, obliquely broadly ovate, acuminate with involute margins, glabrous or externally sparsely ciliate. Lip 5.3–7.0 × 8.0 mm, 3-lobed, basal part reniform with small auricles; middle lobe ca 1/3 f the lip length, triangular-ovate, apiculate, pubescent; disc papillate, with numerus glands along the margin, 5-veined with branching veins. Gynostemium 2.3–2.8 mm long. Figs. 5 and 15.

*Habitat and ecology:* Terrestrial plant found in xerophytic scrub, intermingled with meadows and dryer grassland at the altitude of 3,250 m and in paramo dominated by *Calamagrostis* Adans. (Poaceae) and *Espeletia pycnophylla* Cuatrec. (Asteraceae) at 3,250–3,800 m. Flowering occurs in May and July.

*Notes: Pterichis habenarioides* resembles *P. parvifolia* from which it differs by the lip margin being ornamented with numerus glands along the margin (vs. lip with knob-like projections in *P. parvifolia*). This species is also similar to Colombian endemic *P. colombiana* from which it differs by the glabrous sheaths on the scape, as well as by the petals form (falcate in the middle part) and the dorsal sepal size (much larger than the lateral sepals; Morales, 1986). *Pterichis habenarioides* Schtr. differs from *P. triangularilabia* Kolan. & Szlach. by transversely triangular-elliptic lip with triangular to ligulate apex.

*Representative specimens:* Ecuador. Prov. Azuay. Paramo de Tinajillas between Cuenca and Oña. 27 July 1982. *S.E. Clemants et al. 2200* (NY!, QCA!); Km 67 S of Cuenca on Panamerican Highway. Alt. 3,250 m. 4 May 1973. *L.B. Holm-Nielsen et al. 4985* (AAU!). Prov. Carchi. San Pedro de Huaca. Estación Biológica Guandera. Parroquia Marsical Sucre. Alt. 3,500 m. 12 July 2000. *E. Narváez 586* (QCNE!). Prov. Imbabura. Timber line vegetation on Hacienda Yura Cruz. 10 km N of Ibarra. Alt. 3,700–3,800 m. 25 May 1973. *L.B. Holm-Nielsen et al. 6492* (AAU!).

***Pterichis multiflora*** (Lindl.) Schltr., Bot. Jahrb. Syst. 45: 389. 1911. *Acraea multiflora* Lindl., Orchid. Linden.: 26. 1846. TYPE (Garay, 1978: 185): Venezuela. Mérida. *J. Linden 673* (lectotype K-L, K—Garay’s illustration!, isolectotypes: G-00169178!, P?).

Plants 13–55 cm tall. Leaf petiolate; petiole 4.0–12.0 cm long; blade 7.0–14.0 cm long, up to 3.5 cm wide, elliptic to oblong-lanceolate, subobtuse. Scape glandular-ciliate above basal sheath or from the base, enclosed with 3–7 sheaths, the uppermost ciliate. Inflorescence 3.0–18.0 cm long, subdensely 5–20-flowered, rachis densely ciliate. Flowers with dirty yellowish-green or dirty brown-green sepals with darker greenish-brown veins, petals brown or reddish brown in the basal third, pale yellowish-brown in the center and yellowish at the apex, lip brownish yellow with dark brown veins. Floral bracts 7.1–11.0 mm long, ovate-lanceolate, acuminate, densely glandular-pubescent. Pedicellate ovary 11.0–18.0 mm long, pubescent. Dorsal sepal 7.1–9.0 × 2.3–3.4 mm, ovate-lanceolate, obtuse to acuminate, 3- or 5-veined, externally ciliate. Petals 7.2–10.1 × 1.2–2.3 mm, agglutinate to dorsal sepal, linear-oblong to almost filiform, 2- or 3-veined, glabrous. Lateral sepals 5.2–11.0 × 2.7–5.0 mm, oblique, ovate-lanceolate to ovate, obtuse to acuminate, 3-veined, externally ciliate. Lip 4.2–8 × 7–10 mm, obscurely 3-lobed, basal part reniform above truncate base; middle lobe ca ½ of the lip length, rather large in proportion, ovate-triangular, recurved, densely papillose-hirsute; disc papillate with numerous glands along margin. Gynostemium 2.1–3.5 mm long.

*Habitat and ecology:* Rupicolous, in grass páramo and virgin elfin forest as well as on slopes along road in cloud forest at the altitudes of 2,700–3,850 m. Flowering occurs in January, March, May, June, July, and August.

*Notes:* This species is similar to *Pterichis triloba* which, however, has oblong-elliptic to oblong-lanceolate petals widened near the middle. The 2–3-veined petals makes *P. multiflora* somewhat similar to *P. triangularilabia* and *P. habenarioides* but in *P. multiflora* petals are 5–6 times longer than wide (vs. 3–4 times longer than wide). The illustration of type specimen deposited in K was made based on plant deposited in the same herbarium, however, we did not find this specimen among herbarium sheets.

Collection *Linden 673* deposited in P herbarium (P00363510) represents not *Pterichis* but *Ponthieva*.

*Representative specimens:* Ecuador. Prov. Azuay. Páramo de Tinajillas, km 23-28 from Cumbe on road to Loja. Alt. 3,200–3,300 m. 16 June 1979. *B. Løjtnant et al. 14951* (AAU!). Prov. Cotopaxi. Latacunga-Quevedo road, 3–5 km above Pilaló. Alt. 2,700–2,800 m. 28 May 1979. *B. Løjtnant & U. Molau 13910* (AAU!); Volcán Cotopaxi. Along the road to and at Limpio Punga. Alt. 3,500–3,850 m. 23 March 1984. *S. Laegaard 51851* (AAU!). Prov. Morona-Santiago. Along road from Cuenca to Limon. Alt. 3,000 m. Jan 1989. *C. Luer, J. Luer, A. Andreetta, P. Jesup & S. Ortega sub A. Hirtz 4171* (RPSC!, UGDA-DLSz!—drawing). Prov. Pichincha. Mt. Pichincha, above Quito along road to the crest. Alt. 3,100 m. Aug 1985. *A. Hirtz & W. Flores 2614* (RPSC!). Prov. Sucumbíos. Alt. ca. 3,300 m. 6 July 2017. *M. Kolanowska et al. E17/39* (photo!).

The following collections correspond to *P. multiflora* but the plants have glabrous floral bracts. In our opinion they should be considered as a separated variety.

**Figure 16:**
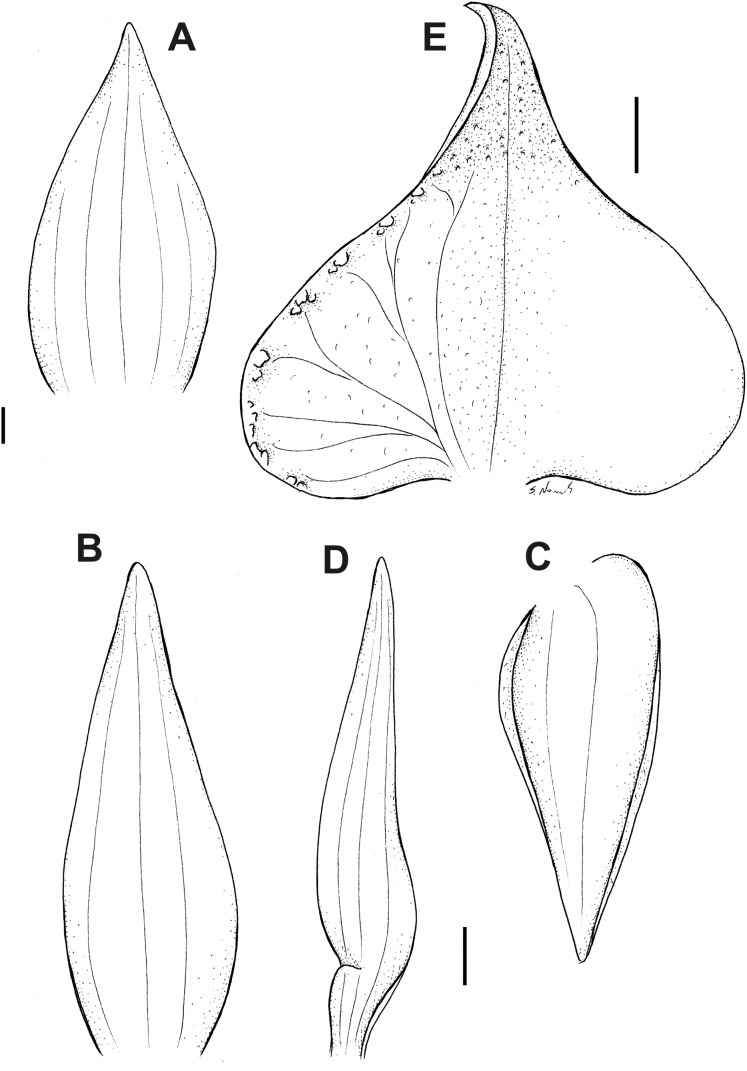
*Pterichis multiflora* var. *ecuadorensis* Kolan., Szlach. & S. Nowak, *var. nov*. (A) Floral bract. (B) Dorsal sepal. (C) Lateral sepal. (D) Petal. (E) Lip. Scale bars = 1 mm. Drawn by S. Nowak from *Madsen 86746* (AAU).

**Figure 17:**
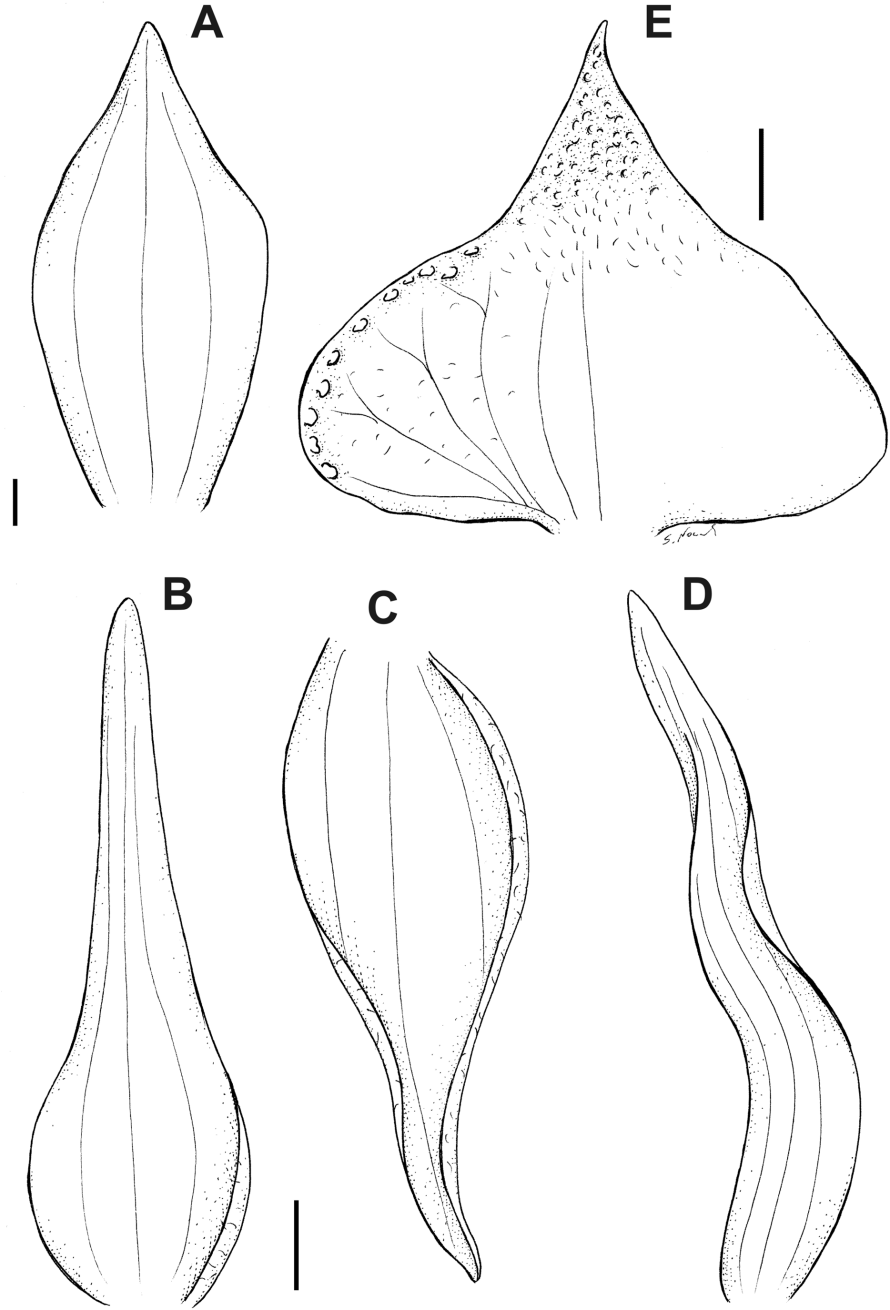
*Pterichis multiflora* var. *ecuadorensis* Kolan., Szlach. & S. Nowak, *var. nov*. (A) Floral bract. (B) Dorsal sepal. (C) Lateral sepal. (D) Petal. (E) Lip. Scale bars = 1 mm. Drawn by S. Nowak from *Clark & Fair 3459* (QCNE).

***Pterichis multiflora* var. *ecuadorensis*** Kolan., Szlach. & S. Nowak, ***var. nov*.** TYPE: Ecuador. Prov. Tungurahua. Ambato Cantón. Parque Nacional Llanganates. Cordillera de los Llanganates. Laguna de Soguillas. Páramo. Perianth mostly yellow with maroon streaks. Alt. 3,700 m. 27 November 1996. *J.L. Clark & J. Fair 3459* (QCNE!). Figs. 5, 16 and 17.

*Distinguished from typical form of Pterichis multiflora by glabrous floral bracts*.

*Etymology*: In reference to the geographical distribution of the new variety.

*Representative specimens:* Ecuador. Prov. Azuay. km 50 S of Cuenca on Panamerican highway. Páramo. Alt. ca. 3,500 m. 6–10 May 1973. Terrestrial in grass paramo. *L.B. Holm-Nielsen et al. 5128* (AAU!). Prov. Carchi. Páramo El Angel. In the pass on road El Angel-Tulcán. Very humid *Espeletia-*páramo. Alt. 3,750–3,850 m. 15 May 1973. *L.B. Holm-Nielsen et al. 5450* (AAU!). Prov. Loja. Road Pichig-Fierro Urc, ca. km 10. Paramo and upper montane forest. Alt. 3,000–3,500 m. 20 Jan 1990. *J.E. Madsen 86746* (AAU!). Prov. Napo. Cordillera de los Llanganates, Loma between Río Topo and Río Verde Grande. 3 km NW of Cerro Hermoso. Alt. 4,000 m. 10 November 1980. *L.B. Holm-Nielsen et al. 28327* (AAU!). Prov. Tungurahua. Ambato Cantón. Parque Nacional Llanganates. Cordillera de los Llanganates. Laguna de Soguillas. Páramo. Perianth mostly yellow with maroon streaks. Alt. 3,700 m. 27 November 1996. *J.L. Clark & J. Fair 3459* (QCNE!).

***Pterichis parvifolia*** (Lindl.) Schltr., Bot. Jahrb. Syst. 45: 389. 1911. ≡ *Acraea parvifolia* Lindl., Pl. Hartw.: 155. 1845. TYPE (Garay, 1978: 186): Ecuador. Loja. *Hartweg 50* (lectotype K-L, K-L-Garay’s illustration!).

*Pterichis barbifrons* (Kraenzl.) Schltr., Repert. Spec. Nov. Regni Veg. Beih. 9: 127. 1921. ≡ *Prescottia barbifrons* Kraenzl., Bot. Jahrb. Syst. 54, Beibl. 117: 19. 1916. TYPE (Garay, 1978: 186): Peru. *A. Weberbauer s.n*. (lectotype B; AMES-00103619!—photo, F-0BN018368!—photo).

**Figure 18:**
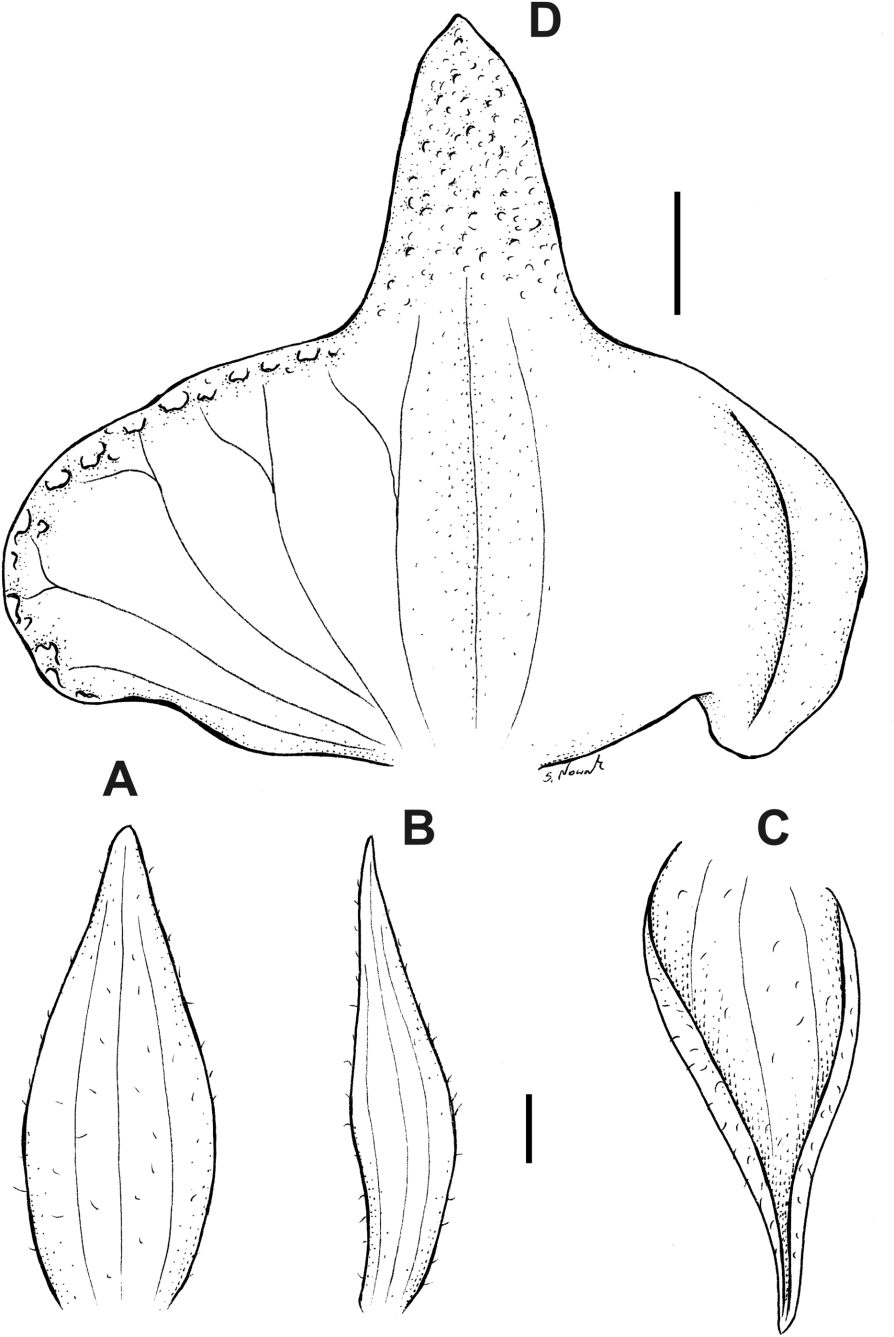
*Pterichis parvifolia* (Lindl.) Schltr. (A) Dorsal sepal. (B) Lateral sepal. (C) Petal. (D) Lip. Scale bars = 1 mm. Drawn by S. Nowak from *Øllgard & al. 34471* (AAU).

Plant 18–45 cm tall. Leaf petiolate; petiole 5.0–6.0 cm long, canaliculated; blade 5.4–7.0 × 1.4–2.0 cm, ligulate to narrowly elliptic, subacute. Scape pubescent, with 4–5 sheaths decreasing in size distally along the scape, pubescent in upper half. Inflorescence 3.5–7.0 cm long, subdensely 7–10-flowered, rachis pubescent. Flowers greenish or brownish with yellow lip with brownish veins. Floral bracts 6–12 mm long, ovate-lanceolate, acute, glabrous. Pedicellate ovary 10.5–21 mm long, pubescent. Dorsal sepal 8.0–9.0 × 2.3–3 mm, ovate-lanceolate, subacuminate, obtuse, 3-veined, externally sparsely ciliate. Petals 8–10 × 1.5–2.5 mm, agglutinate to dorsal sepal, unguiculate in lower fifth, obliquely oblong-lanceolate, subacute, 3-veined, ciliate along margin. Lateral sepals 8.1–8.5 × 3.6–4.0 mm, obliquely ovate, subacuminate, 3- or 4-veined, externally sparsely ciliate. Lip 5.1–5.5 × 9.5–10.0 mm, 3-lobed, basal part reniform above truncate base, with small auricles; middle lobe shorter than lip half length, ovate-triangular, densely glandular-pubescent; disc ornamented with a knob-like projections along margin, apical margin ciliate, 7-veined with branching veins. Gynostemium 3.0–4.0 mm long. Figs. 5 and 18.

*Habitat and ecology:* Terrestrial in very humid montane forest, in paramo and on steep mossy exposed slopes without trees at the altitudes of 3,200–3,800 m. Flowering occurs in June, July and August.

*Notes:* This species differs from similar *P. habenarioides* by the obliquely oblong-lanceolate (vs. obovate-lanceolate) petals, glabrous (or almost glabrous) sepals (vs. sepals pubescent), and a presence of a single row of the large papillae along the lip margin (vs. papillae small, arranged in 2–3 rows). *Pterichis barbifrons* and it was not accepted by most subsequent researchers as separated species (Schweinfurth, 1958; Garay, 1978). Unfortunately the type of *P. barbifrons* was lost and the actual concept of this taxon is confusing. It was considered as synonym of *P. galeata* by Schweinfurth (1941) and this concept was followed by Kolanowska & Szlachetko (2017), however, more detailed studies of available literature indicated that *P. barbifrons* has glabrous floral bracts and its petals are adnate to the dorsal sepal. Hereby this species fits more the morphological characteristic of *P. parvifolia* as proposed by Garay (1978).

*Representative specimens:* Ecuador. Prov. Azuay. Between Cumbe and El Progreso. Alt. 3,200 m. 4 Aug 1975. *C. Luer et al. 411* (RPSC!, UGDA-DLSz!—drawing). Prov. Loja. *C.T. Hartweg 50* (K-L-Garay’s illustration!). Prov. Napo. Quijos. Sector de Papallacta. Sendero hacia las Lagunas de Cojunco y Verde. Alt. 3,350–3,450 m. Bosque muy humedo subalpino, paramo herbaceo. 25 June 2000. *A. Álvarez et al. 2613* (QCNE!). Prov. Pichincha/Napo. Road Olmedo-Laguna San Marcos, E of the pass. Alt. 3,620–3,800 m. 10–11 July 1980. *B. Øllgard et al. 34471* (AAU!).

***Pterichis pauciflora*** Schltr., Repert. Spec. Nov. Regni Veg., Beih. 8: 41. 1921. TYPE (Garay, 1978: 186): Ecuador. Loja. In Andibus orientalibus. Alt. 3,000–3,400 m. *F. Lehmann 7111* (B†; lectotype K-000573776!, isolectotype AMES-00083571—fragment).

**Figure 19:**
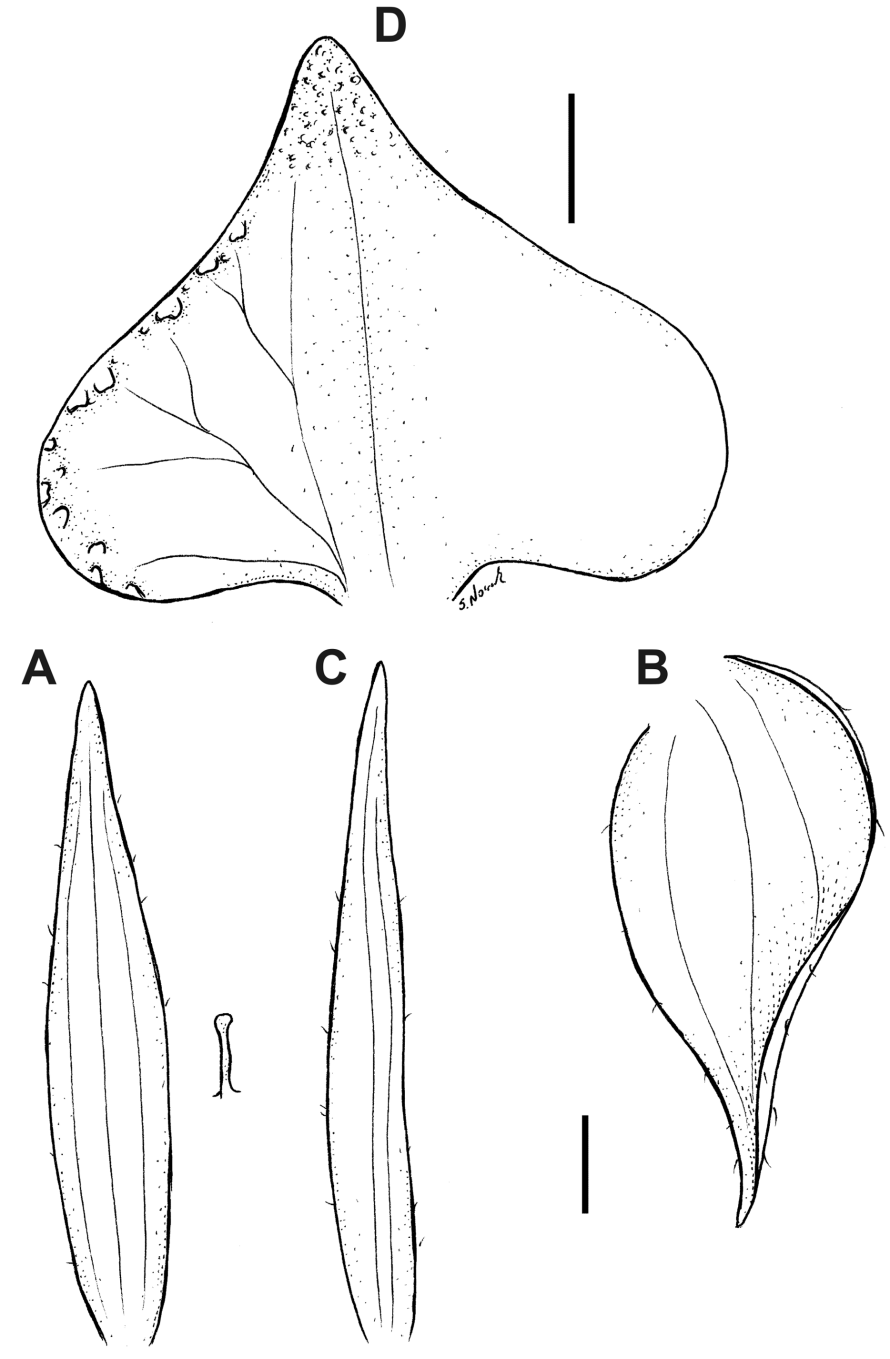
*Pterichis pauciflora* Schltr. (A) Dorsal sepal. (B) Lateral sepal. (C) Petal. (D) Lip. Scale bars = 1 mm. Drawn by S. Nowak from *Holm-Nielsen al. 17247* (AAU).

Plant 14–40 cm tall. Leaf petiolate; petiole 5.0–8.0 cm long; blade 4.0–10.0 cm × 1.2 cm, linear to oblong-lanceolate, acute. Scape minutely puberulent in the upper part, with 4–5 tubular, pubescent sheaths. Inflorescence 3.0–7.0 cm long, 2–12-flowered, rachis densely ciliate. Flowers yellowish-orange to yellowish-brown, with brown veins. Floral bracts up to 8.0 mm long, ovate-lanceolate, glabrous. Pedicellate ovary 8–12 mm long, densely glandular. Dorsal sepal 5–8 × 1.3–3 mm, lanceolate-ovate to ovate, obtuse, 3-veined, glabrous or externally very sparsely ciliate. Petals 5.2–7.8 × 1–2.2 mm, agglutinate to dorsal sepal, obliquely linear-lanceolate, acuminate, l- or 2-veined, margin sparsely ciliate. Lateral sepals 5–6 × 2.8–4.5 mm, obliquely ovate, acuminate, 3−veined, glabrous or externally sparsely ciliate. Lip 4.5–7.5 × 5.5–8.5 mm, basal part reniform above truncate base; middle lobe indistinctive, triangular, obtuse, glandular-papillose; disc 7-veined, margin ornamented with large glands. Gynostemium 2.5–3.2 mm long. Figs. 5 and 19.

*Habitat and ecology:* Terrestrial in open, humid places in high mountains and in paramo dominated by *Blechnum*, *Elaphoglossum* Schott *ex* J.Sm. (Lomariopsidaceae), *Monticalia* (=*Senecio*) *andicola* (Turcz.) C.Jeffrey (Asteraceae), at the altitudes of 3,000–3,910 m. Flowering occurs in February and April.

*Notes:* The lip basal part of *Pterichis pauciflora* is not well-separated from the middle lobe unlike in the most species of section *Acraea*. From somewhat similar *P. habenarioides* and *P. parvifolia* it differs also in having usually 1-veined petals. Unlike *P. habenaroides* this species has linear-lanceolate petals (Schlechter, 1921). In somewhat similar *P. triloba* petals are sparsely ciliate on margin and 3-veined.

*Representative specimens:* Ecuador. Prov. Azuay. Cuenca Sayaúsi. Parque Nacional Cajas, cerca del sector San Luis. Alt. 3,910 m. 5 February 2013. *D. Minga & A. Verdugo 2530* (HA 8413!). Prov. Loja. In Andibus orientalibus. Alt. 3,000–3,400 m. *F. Lehmann 7111* (K-000573776!). Prov. Napo. N side of Cerro Sumaco, upper part of the loma NW od campsite. Humid páramo in furrow-like quebradas dominated by *Blechnum*, *Elaphoglossum* and *Senecio andicola*. Alt. 3,750 m. 25 April 1979. *L.B. Holm-Nielsen et al. 17247* (AAU!).

***Pterichis seleniglossa*** Schltr., Repert. Spec. Nov. Regni Veg. Beih. 8: 42. 1921. TYPE: Ecuador. Pichincha. Ad rupes montium. Alt. 3,300 m. *L. Sodiro s.n*. (B?).

**Figure 20:**
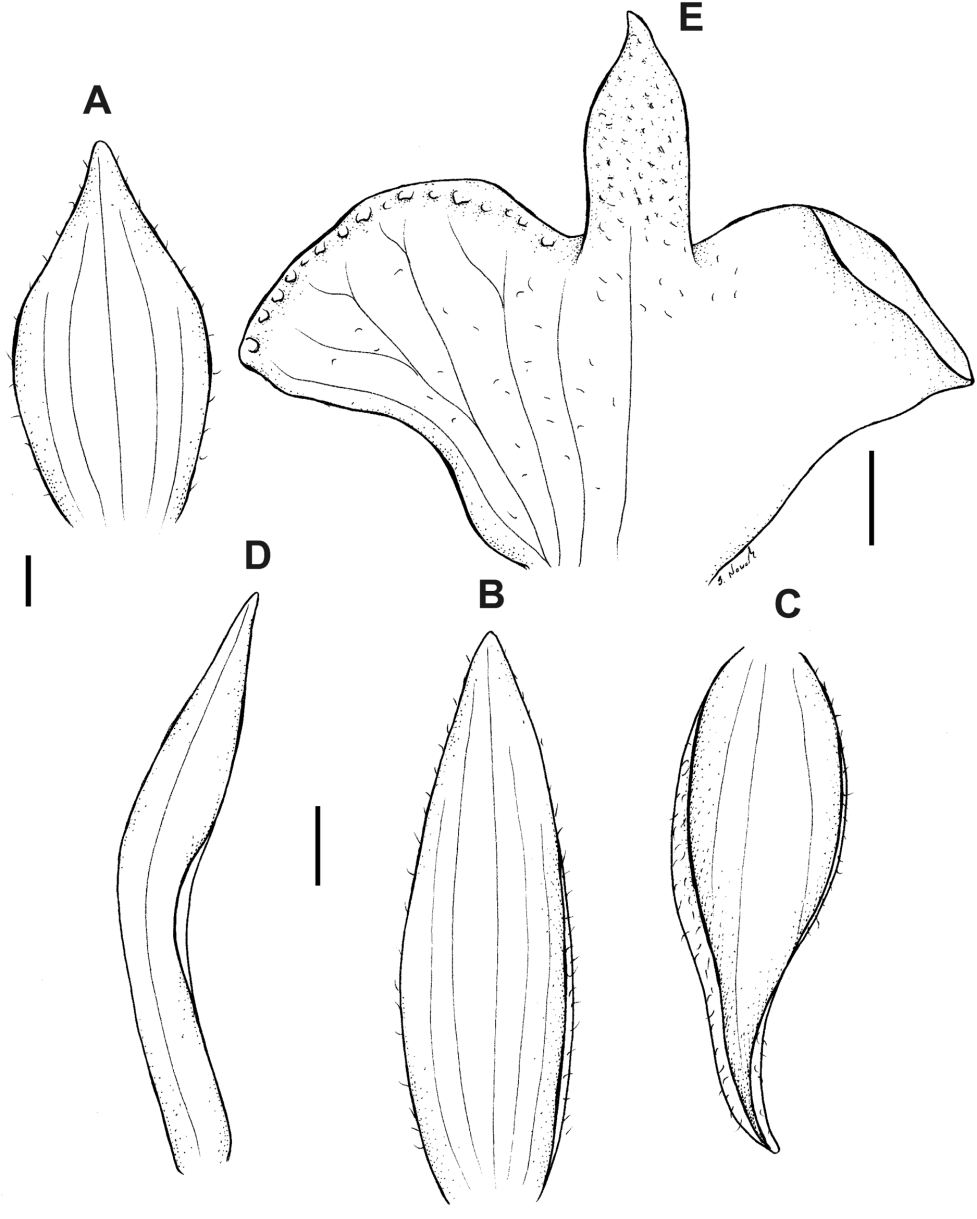
*Pterichis seleniglossa* Schltr. (A) Floral bract. (B) Dorsal sepal. (C) Lateral sepal. (D) Petal. (E) Lip. Scale bars = 1 mm. Drawn by S. Nowak from *Dodson & al. 16396* (QCNE).

Plant 16–35 cm tall. Leaf petiolate; petiole 2.0–10.0 cm long; blade 5.0–16.0 × 1.5–2.3 cm, linear to oblong-lanceolate, acute. Scape, minutely puberulent in the upper part, with 3–6 tubular, glandular sheaths. Inflorescence 5.0–7.5 cm long, sublaxly to subdensely 8-13-flowered; rachis densely ciliate. Flowers with dark green or brown sepals and petals and brown or yellow lip marked with red-brown or yellow. Floral bracts 7.0–8.0 mm long, ovate-lanceolate, glandular-ciliate. Pedicellate ovary 8.0–12.0 mm long, densely glandular-ciliate. Dorsal sepal 6.0–9.0 × 1.6–3.2 mm, ovate-lanceolate to ovate, obtuse, 3- or 5-veined, externally ciliate. Petals 6.5–9.0 × 1.7–2.2 mm, agglutinate to dorsal sepal, obliquely linear-lanceolate or linear, acuminate, obtuse, l-veined, glabrous. Lateral sepals 6.0–8.0 × 2.1–3.5 mm, obliquely ovate-lanceolate to ovate, obtuse, 3-veined, externally ciliate. Lip 3.2–6.8 × 7.5–9.0 mm, basal part lunate above truncate base; middle lobe shorter than lip half length, elliptic, obtuse, papillate; disc with numerous small glands along the margin, 7- or 9-veined, veins apically branching. Gynostemium 2.5–3.0 mm long. Figs. 5 and 20.

*Habitat and ecology:* Terrestrial in high-montane forest and along roadside in grass paramo at the altitudes of 3,050–3,300 m. Flowering occurs in March, July and August.

*Notes:* Schweinfurth (1958) and Garay (1978) considered this species synonymous to *Pterichis triloba*. Based on our study the two species differs in petal venation. Petals of *P. seleniglossa* are 1-veined (vs. 3-veined in *P. triloba*). *Pterichis seleniglossa* resembles Bolivian *P. mandonii* Rolfe but in the latter species the lip middle lobe is much shorter.

*Representative specimens:* Ecuador. Prov. Bolivar. Guaranda-Caluma-Catarama, km 7.7. Alt. 3,050 m. 5 Jul 1991. *C.H. Dodson, N. Williams & M. Whitten 18767* (RPSC!, UGDA-DLSz!—drawing). Prov. Pichincha. Yanacocha Reserve. 12 August 2018. *M. Kolanowska, S. Nowak & A. Hirtz E18/14* (photo!), Reserva Gebotánica del Pululahua. Alt. 3,100 m. 1 March 2001. *C. Cerón & E. Freire 43973* (QAP 38931!); Ad rupes montium. Alt. 3,300 m. *L. Sodiro s.n*. (Schlechter, 1921). Prov. Imbabura. Road from Otavalo to Selva Alegre via Lago Cuicocha entering from Panamerican highway between Otavalo and Ibarra. Alt. 3,100 m. 20 March 1986. *C.H. Dodson et al. 16396* (QCNE!).

***Pterichis triloba*** (Lindl.) Schltr., Bot. Jahrb. Syst. 45: 389. 1911. ≡ *Acraea triloba* Lindl., Ann. Mag. Nat. Hist. 15: 386. 1845. TYPE (Garay, 1978: 187): Ecuador. Prov. Pichincha. Hda. de Pinantura, 35 km SE of Quito. *C.T. Hartweg s.n*. (lectotype K-L, K-L-Garay’s illustration!, isolectotype AMES-00082267!—fragment).

**Figure 21:**
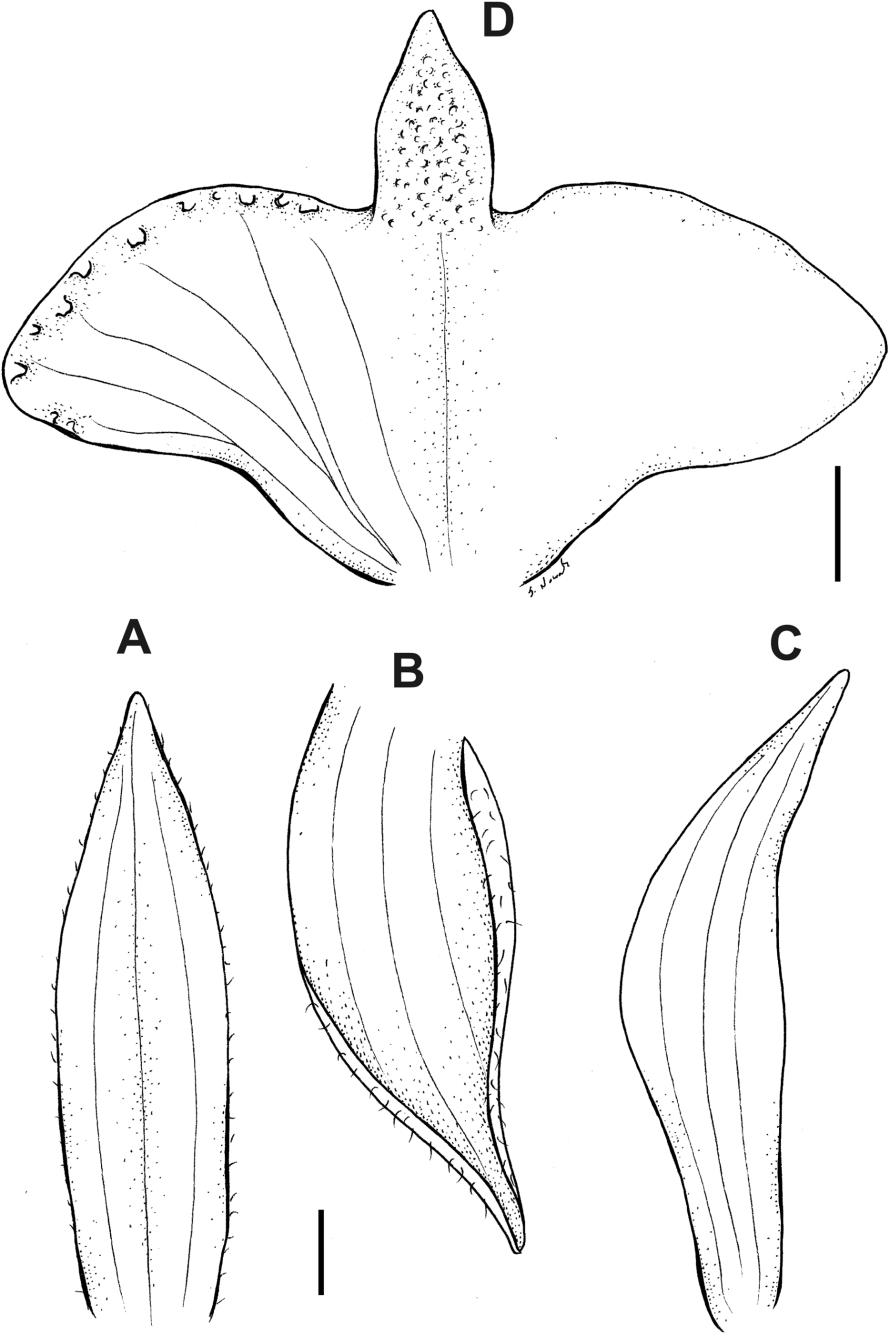
*Pterichis triloba* (Lindl.) Schltr. (A) Dorsal sepal. (B) Lateral sepal. (C) Petal. (D) Lip. Scale bars = 1 mm. Drawn by S. Nowak from *Benoiss 2315* (Q).

Plants 27–34 cm tall. Leaf sessile, (6.0)16.0–24.0 cm long, narrowly lanceolate, acuminate. Scape ciliate in the upper half, enclosed with 4–5 tubular sheaths which are gradually shorter towards the scape. Inflorescence 4.0–8.0 cm long, sublaxly to subdensely 7–15-flowered, rachis ciliate. Flowers with greenish-brown sepals and petals and basally red-brown lip with yellow apex. Floral bracts 7.0–11.0 mm long, ovate or ovate-lanceolate, ciliate. Pedicellate ovary 10.0–14.0 mm long, pubescent. Dorsal sepal 7.0–10.0 × 1.8–3.1 mm, elliptic-lanceolate, obtuse, 3-veined, externally ciliate. Petals 7.0–10.0 × 1.0–3.0 mm, agglutinate to dorsal sepal, oblong-elliptic to oblong-lanceolate, obtuse, 3-veined, glabrous. Lateral sepals 6.0–9.3 × 2.0–3.2 mm, obliquely elliptic-ovate, subacute, 3-veined, externally ciliate. Lip 5.1–6.7 × 7.5–9.0 mm, distinctly 3-lobed, basal part reniform-flabellate above truncate base; middle lobe ca 1/3 of the lip length, elliptic, apiculate; disc glandular with numerous glands along margin. Gynostemium 2.2–4.0 mm long. Figs. 5 and 21.

*Habitat and ecology:* Terrestrial on steep slopes in high mountains and in mossy scrub forest and open grasslands at the altitudes of 3,100–3,600 m. Flowering occurs in March, April, May and July.

*Notes:* This species resembles somewhat *P. tunguraguona* in which the truncate base of the lip is very short (less than 1/3 of the lip lamina vs. 1/3 of the lip lamina) and there are no glands or projections along the margin of the basal lip part.

*Representative specimens:* Ecuador. Prov. Imbabura. Laguna Culcocha, crater lake 30 km W of Ibarra. 3,100 m. 24 May 1973. Terrestrial on a steep rocky slope. *L.B. Holm-Nielsen et al. 6370* (AAU!). Prov. Pichincha. 30 March 1930. *R. Benoiss 2315* (Q 18090!); Quito-Santo Domingo via Chiriboga, km 20. Alt. 3,400 m. 15 May 1981. Terrestrial in paramo. *C.H. Dodson et al. 10837* (Q!, QCA!, RPSC!); Antisana, camino entre Inga Montserrat y Vaquero Pasana. Alt. 3,400–3,600 m. 13 April 1992. *A. Fieire-Fierro et al. 2143* (NY!, QCA!); Mt. Mojanda, at the TV antenna on top of Mt Mojanda entering from km 10 on the Otavalo-Selva Alegre road. Alt. 3,100–3,400 m. 31 July 1985. *C.H. Dodson & A. Embree 16121* (RPSC!, UGDA-DLSz!—drawing); Hda. de Pinantura, 35 km SE of Quito. *C.T. Hartweg s.n*. (K-L – Garay’s illustration!, AMES!); Volcan Corazón. Alt. 3330. 4 July 2017. *M. Kolanowska et al. E17/10* (photo!).

***Pterichis tunguraguona*** Rchb. f. *ex* Szlach. & Kolan., Ann. Bot. Fenn. 51(5): 331. 2014. TYPE: Ecuador. *W. Jameson s.n*. (holotype W 878!).

**Figure 22:**
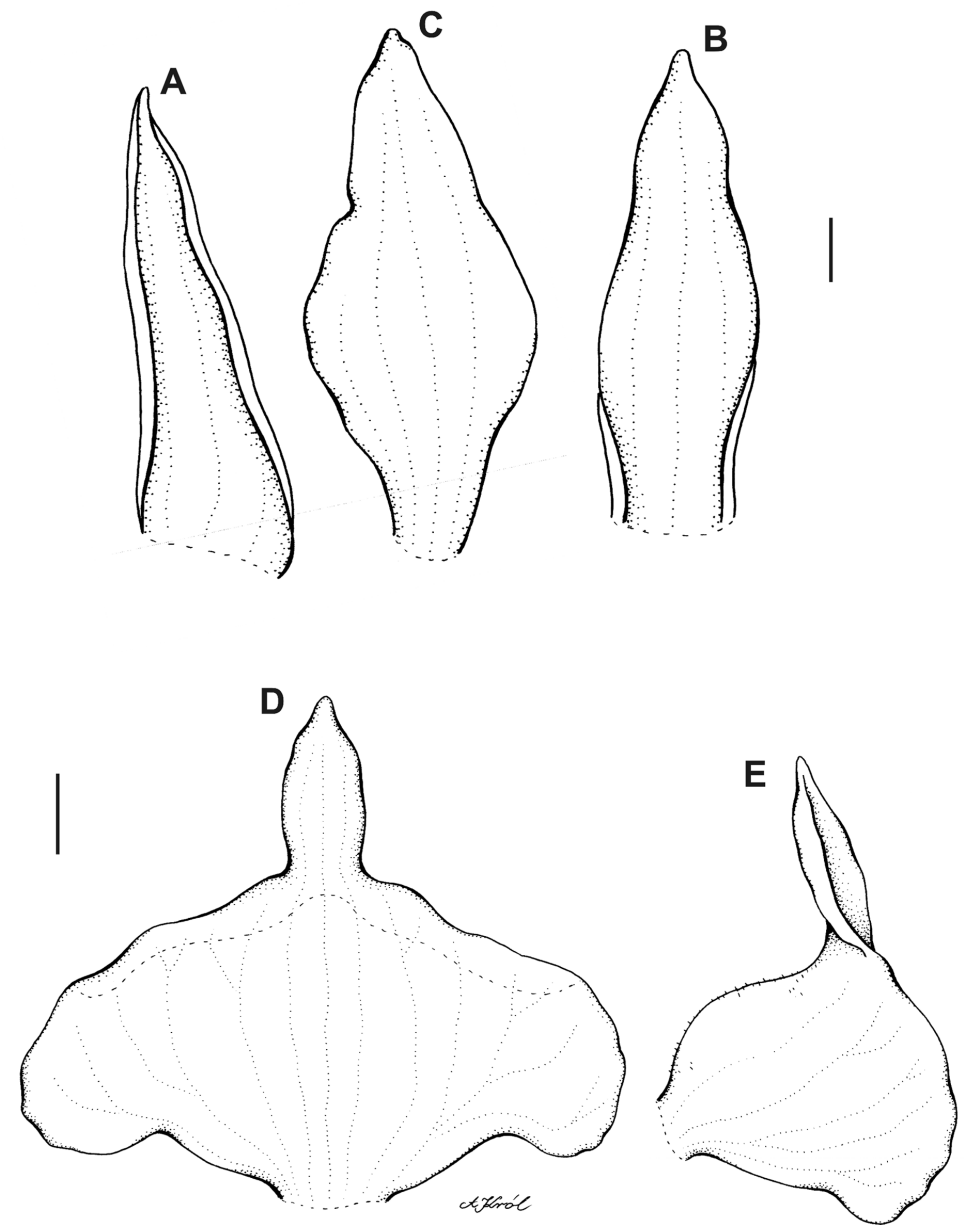
*Pterichis tunguraguona* Rchb. f. *ex* Szlach. & Kolan. (A) Lateral sepal. (B) Dorsal sepal. (C) Petal. (D) Lip, front view. (E) Lip, lateral view. Scale bars = 1 mm. Drawn by A. Król from *Jameson s.n*. (holotype W).

Plant 16–40 cm long. Leaf petiolate; petiole 2.8–7.0 cm long; blade 5.0–14.0 × 1.3–3.0 cm, narrowly elliptic, obtuse to acute. Scape glabrous, enclosed in 3–5 tubular sheaths, the uppermost sheaths often ciliate. Inflorescence 4.5–17.0 cm long, subdensely to sublaxly 7–15-flowered. Flowers white-yellow or olive-green with weak brown-green stipes sepals, yellowish petals which are basally red-brown tinted, and brown-yellow or greenish yellow lip with dark brown stripes, apex pale yellow. Floral bracts 7.0–12.0 mm long, lanceolate to elliptic, sparsely ciliate. Pedicellate ovary 7.0–13.0 mm long, glandular-ciliate. Dorsal sepal 6.1–8.8 × 1.9–2.8 mm, oblong-lanceolate to ovate, obtuse, 3- or 5-veined, externally papillate. Petals 7.0–9.0 × 2–4 mm, agglutinate to dorsal sepal, narrowly ovate above narrow claw, obtuse, 3-veined, glabrous. Lateral sepals 7.0–9.5 × 3.0–3.5 mm, obliquely ovate-lanceolate, obtuse, margins incurved, 3-veined, externally papillate. Lip 5.8–6.5 × 8.0–9.5 mm, basal part reniform-lunate above truncate base, with small auricles; middle lobe ca 1/3 of the lip length, ligulate, papillate; disc papillate, with numerous small glands along the margins, 7- or 9-veined, veins apically branching. Gynostemium 2.2–2.5 mm long. Figs. 5 and 22.

*Habitat and ecology:* Terrestrial in paramo, in montane forest and on the trails at the altitudes of 2,900–3,660 m. Flowering occurs in January, May and September.

*Notes:* This is one of four Ecuadorian representatives of sect. *Acraea* with glabrous petals. It can be distinguished from *Pterichis seleniglossa* and *P. triloba* by relatively short truncate basal part of the lip (less than 1/3 of the lip lamina). *Pterichis tunguraguona* is resembles *P. parviflora* but the petals of *P. tunguraguona* are shortly unguiculate at base (vs. non-unguiculate), narrowly ovate above (vs. narrowly-oblong), distinctly wider than sepals (vs. narrower than sepals). Moreover, its lip is transversely elliptic, semilunate at the base (vs. lip basal part semiorbicular) and papillate along apical margins and on the apex, and the lip apical part is ligulate-lanceolate (vs. triangular). Distal lip margins are papillate and densely ornamented by numerous, swollen outgrowths, which are missing in *P. tunguraguona*.

*Representative specimens:* Ecuador. Prov. Azuay. km 91 on Panamaerican Highway N of Loja. Dry scrub. Alt. 2,900 m. 5 May 1973. *L.B. Holm-Nielsen et al. 5108* (AAU!). Prov. Imbabura. Vía Joya-Laguna de Mojanda Cajas. Al sur de la población de Otavalo. Alt. 3,109–3,659 m. 26 January 1980. *J. Jaramillo & F. Coello 2042* (AAU!, QCA!); Above Piñán, slopes of Volcan de Cotacachi. Alt. 10,450 ft. 3 September 1944. *W. B. Drew E-91* (QCNE!). *Sine loc*. *W. Jameson s.n*. (W!).

***Pterichis hirtziana*** Kolan., Szlach. & S. Nowak, ***sp. nov*.** TYPE: Ecuador. Villonaco. Ca. 15 km W of Loja. Alt. 2,500–2,900 m. 24 April 1987. *H. van der Werff & W. Palacios 8956* (holotype: QCNE 34289!).

*Species similar to Pterichis habenarioides distinguished by lunate basal lip part and obliquely oblong-elliptic petals*.

**Figure 23:**
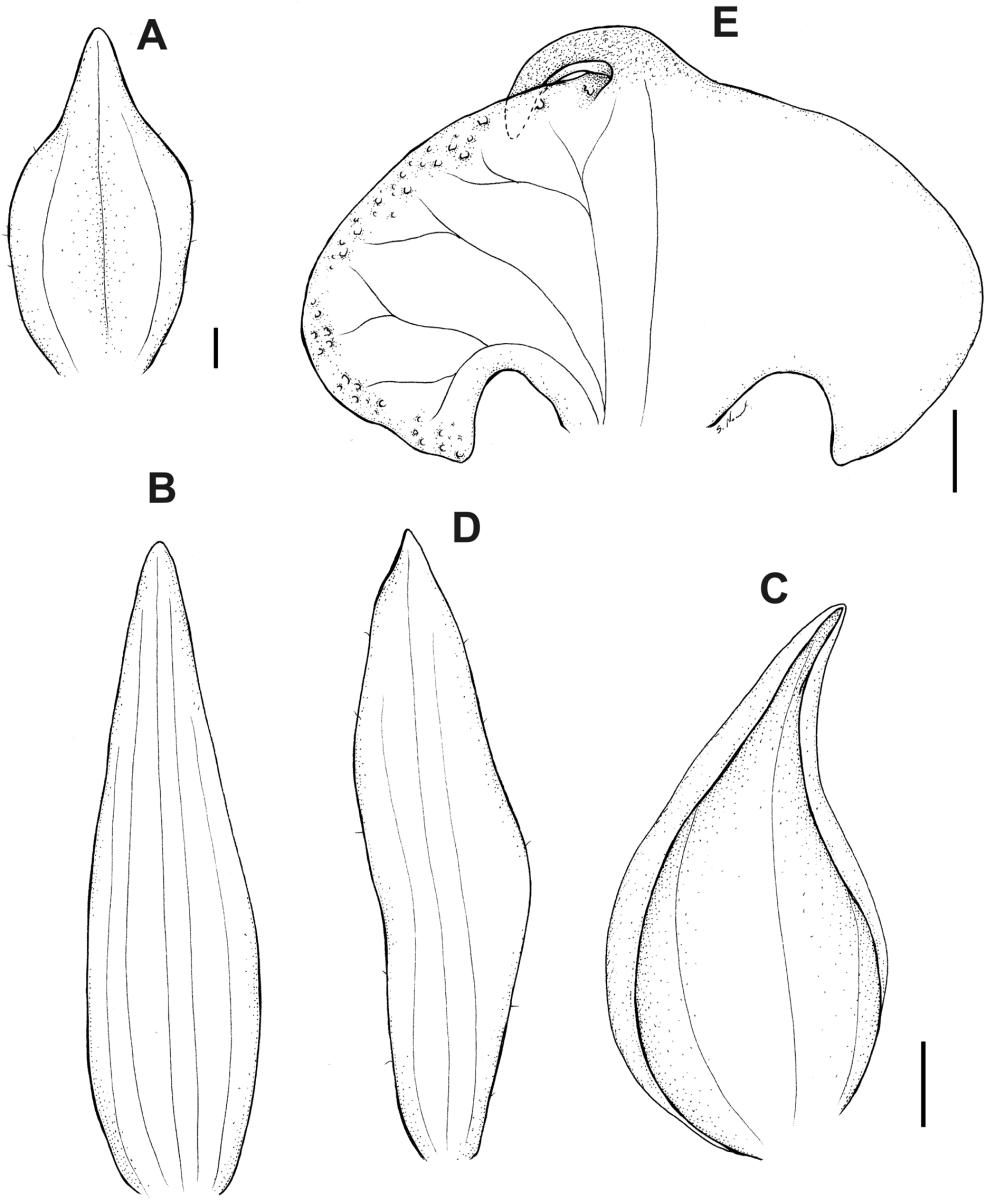
*Pterichis hirtziana* Kolan., Szlach. & S. Nowak, *sp. nov*. (A) Floral bract. (B) Dorsal sepal. (C) Lateral sepal. (D) Petal. (E) Lip. Scale bars = 1 mm. Drawn by S. Nowak from *van der Werff & Palacios 8956* (QCNE).

Plants about 25 cm tall. Leaf not observed in herbarium material. Scape glandular in the upper part, enclosed in 6 tubular sheaths. Inflorescence 6.0 cm long, 12-flowered, rachis densely ciliate. Flowers greenish-red. Floral bract 8.2 mm long, very sparsely ciliate. Pedicellate ovary 11.0 mm long, ovate to broadly ovate, ciliate. Dorsal sepal 8.3 × 2.2 mm, oblong-ovate, rounded at the apex, 5-veined, glabrous. Petals 8.1 × 1.8 mm, agglutinate to dorsal sepal, obliquely oblong-elliptic, obtuse, 3-veined, sparsely ciliate. Lateral sepals 7.1 × 3.0 mm, broadly ovate, acuminate, subobtuse, 3-veined, glabrous. Lip 5.5 × 8.8 mm, basal part lunate above truncate base; middle lobe ca 1/3 of lip length, oblong-ovate, obtuse, densely papillate; disc sparsely papillate, ornamented with numerous small glands along margins, 7-veined, veins apically branching. Gynostemium 3.0 mm long. Figs. 5 and 23.

*Etymology*: Dedicated to Alexander Hirtz, an eminent Ecuadorian orchidologist.

*Habitat and ecology:* Terrestrial in cloud forest changing into shrubby paramo at the altitudes of 2,500–2,900 m. Flowering occurs in April.

*Notes:* This species resembles *Pterichis habenarioides* in having sparsely ciliate floral bracts, glabrous sepals and sparsely ciliate petals, but it is easily distinguished from the latter by having lunate lip basal part (vs. reniform), and obliquely oblong-elliptic petals (vs. lanceolate).

*Representative specimen:* Ecuador. Prov. Loja. Villonaco. Ca. 15 km W of Loja. Alt. 2,500–2,900 m. 24 April 1987. *H. van der Werff & W. Palacios 8956* (QCNE! 34289).

***Pterichis dodsoniana*** Kolan., Szlach. & S. Nowak, ***sp. nov*.** TYPE: Ecuador. Prov. Azuay. Km 85 on Panamerican highway N of Loja. Alt. 2,850–2,950 m. 3 May 1973. *L.B. Holm-Nielsen et al. 4767* (holotype AAU!; isotype AAU!).

*Species similar to Pterichis habenarioides but with almost linear petals which are longer than dorsal sepal and prominent lip middle lobe*.

**Figure 24:**
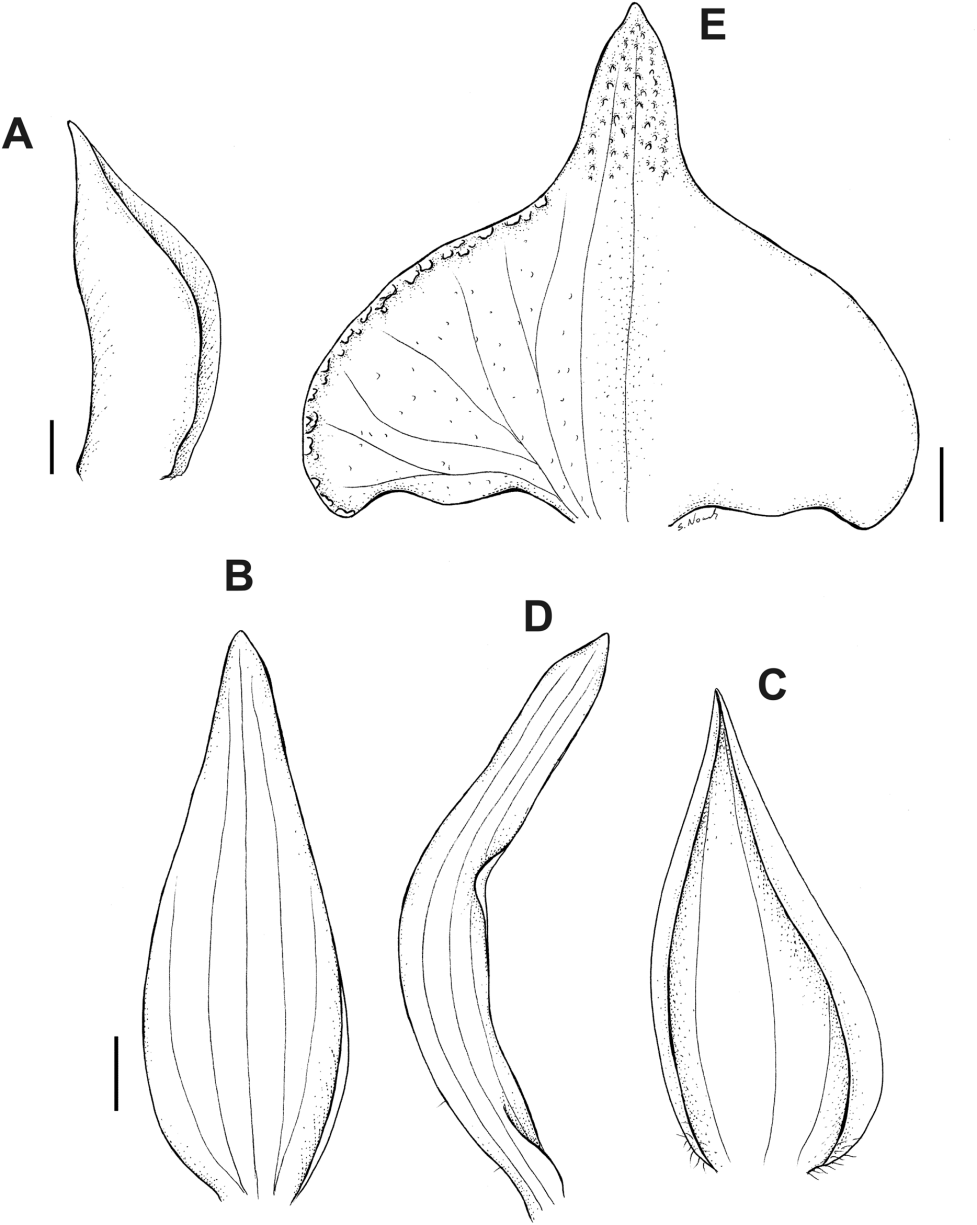
*Pterichis dodsoniana* Kolan., Szlach. & S. Nowak, *sp. nov*. (A) Floral bract. (B) Dorsal sepal. (C) Lateral sepal. (D) Petal. (E) Lip. Scale bars = 1 mm. Drawn by S. Nowak from *Holm-Nielsen & al. 4767* (AAU).

Plants 24–29 cm tall. Leaf petiolate; petiole 7.5 cm long; blade 6.8 cm long, narrowly lanceolate. Scape ciliate above basal sheath, with 3–4 tubular sheaths, the upper one sparsely ciliate. Inflorescence 4.0–10.0 cm long, 4–12-flowered, rachis densely ciliate. Flowers greenish-yellow with green veins, petals yellow with brown veins. Floral bract 6.6 mm long, glabrous. Pedicellate ovary 10.0 mm long, ovate to broadly ovate, pubescent. Dorsal sepal 7.8 × 2.8 mm, narrowly ovate, obtuse, 5-veined, glabrous. Petals 8.2 × 1.2 mm, agglutinate to dorsal sepal, linear, widened in the middle, obtuse, 3-veined, sparsely ciliate. Lateral sepals 6.3 × 3.1 mm, ovate, acuminate, subacute, 3-veined, externally ciliate only near the base. Lip 7.0 × 8.7 mm, basal part reniform above truncate base, with small auricles; middle lobe ca 1/3 of the lip length, ligulate-ovate, apiculate, obtuse, densely papillate; disc sparsely papillate, ornamented with numerous glands along margins, 7-veined. Gynostemium 3.2 mm long. Figs. 5 and 24.

*Etymology*: Dedicated to Dr. Calaway Dodson (1928–2020), eminent orchid taxonomist, who described numerous orchid species from Ecuador. He was the author of partial treatments of Orchidaceae for the Flora of Ecuador project of Gothenburg University (Sweden) and the Universidad Catolica of Ecuador.

*Habitat and ecology:* Terrestrial in dry, low scrub vegetation at the altitudes of 2,850–2,950 m. Flowering in May.

*Notes:* This species resembles *Pterichis habenarioides* in having glabrous floral bracts and sepals and in sparsely ciliate petals, but it is easily distinguished from the latter by having linear petals slightly widened in the middle (vs. lanceolate) which are longer than dorsal sepal, and prominent lip middle lobe constituting more than 1/3 of the total lip length (vs. less than 1/4 of the total lip length).

As holotype serves specimen in the middle part of herbarium sheet, two other plants are therefore isotypes.

*Representative specimen:* Ecuador. Prov. Azuay. km 85 on Panamerican highway N of Loja. Alt. 2,850–2,950 m. 3 May 1973. *L.B. Holm-Nielsen et al. 4767* (AAU!).

***Incertæ sedis***

Material incomplete or too damaged for identification

Ecuador. Prov. Azuay. Cantón Sigsig. Parroquia Jima, parte alta de Moya, sector Zhurugiña. Alt. 3,400 m. *L. Suin et al. 1041* (HA!). Prov. Carchi. Cantón Mantufar, San Gabriel, Loma Bretaña, entre Río Minas y la quebrada Central. Alt. 3,100–2,500 m. 18 August 2010. *C. Cerón et al. 68580* (QAP!). Prov. Cotopaxi. Cantón Sichchos, sector Monte Cimaurcu. Alt. 4,030 m. 2 June 2012. *C. Cerón & D. Jácome 71627* (QAP!). Prov. El Oro. Chilla Cantón. Cerro de Chilla. Vegetación de paramo, arbustiva y herbacea. Alt. 3,595 m. 3 May 1997. *H. Vargas & C. Conaday 1393* (QCNE!). Prov. Imbabura. Alturas de Cayachupa, ca 5 km NW of the village Piñan. Alt. 3,200 m. 16 June 1980 *C. R. Sperling & R. Bleiweiss 5105* (QCA!). Prov. Loja. Parque Nacional Podocarpus. Cerro Toledo. 1 December 1988. *J. E. Madsen & al 75700* (AAU!). Prov. Napo. Parque Nacional Llanganates. Vía Salcedo-Tena, sector Siete vueltas. Alt. 3,600–3,850 m. 26 February 2015. *A.J. Pérez et al. 8317* (QCA!). Prov. Pichincha, cantón Quito. Reserva Geobotánica del Pululahua, sector Lulumbamba. Alt. 2,700 m. 7 June 2008. *C. Cerón & C. Reyes 62369* (QAP!), Cantón Quito. Reserva Geobotánica del Pululahua, Cerro Padre Rumi. Alt. 2,825 m. 2 June 2007. *C. Cerón & C. Reyes 59179* (QAP!). Prov. Zamora-Chinchipe. Cordillera de Sabanilla, cerca de la carretera Jimbura-Zumba. Alt. 3,300–3,500 m. 22 October 1996. *RBu & SL 1221* (QCA!). Without specific locality. Ad rupes Cotursi – Pifi. Alt. 3,000 m. *A. Mille 31* (QPLS!).

## Discussion

Globally orchids are among the most threatened plants due to increase of anthropogenic pressures, high level of endemism and complex relationships with mycorrhizal fungi and pollinators (Wraith, Norman & Pickering, 2020). As suggested by Pillon & Chase (2007), taxonomic recognition of orchids is crucial for planning any conservation actions to protect these plants and species inventories, especially in the most biodiverse tropical areas, are required establish adequate conservation projects (May, 1988; Dubois, 2003). The systematic surveys are particularly important considering the crisis of biodiversity (Ramade, 1999) which can lead to extinction of numerous rare organisms. Unfortunately, also the taxonomic research are in crisis (Tillier, 2000; Dubois, 2003). The lack of clear characteristic of particular species results in numerous misidentifications of known taxa and false local species checklists (Dubois, 2003).

According to our findings only floral bracts and perianth segments are diagnostic characters that allow to identify *Pterichis* species, however, the importance of several characters requires further studies—the genetic analyses could help resolve some classification doubts. For example we found that *P. acuminata* is characterized by a significant variation and we are not able to clearly state that small deviations from the typical morphology of the floral segments are enough to recognize some populations as separated species (see notes in *P. acuminata*). The ornamentation of floral bracts seems to be consistent within the same species and the bracts are either ciliate or glabrous. Rarely intermediate form of floral bract, with just few ciliae, was observed. The similar scheme was recorded for petals and sepals which shows the same pattern of ornamentation within the species (but see notes in *P. acuminata*). The lip seems to be the most conservative character—its form, relative size and distribution of papillae or knobs is consistent in populations of the same species. The venation of tepals is the most disputable diagnostic character and it requires further analyses. While usually petals of *Pterichis* representatives are 1- or 3-veined and sepals are 3- or 5-veined, some populations with 2- or 4-veined petals were found during our research. Our new species are described based on several (at least three) differences between novelties and the most similar taxa.

In this article the new species are described based on a single or just several plants of the same collection, however, it is not an unusual situation. Preferably any new taxon should be formally named after the examination of numerous randomly collected specimens from various populations (Dubois, 2010). Nevertheless, a considerable number of species, not only plants, are described based on a single specimen (Captain et al., 2019; Wells, Johanson & Dostine, 2019). The disadvantage of using a few specimens for the description of new taxa, particularly of new species, is that the intraspecific variation cannot be evaluated. As calculated by Raven et al. (2020) an average of 749 new vascular plant species has been described annually from Latin America for the past 25 years and this rate did not decrease with time. According to some estimates (Bebber et al., 2010) about 70,000 species of flowering plants are waiting to be discovered and named. Around half of the anticipated missing species presumably have already been collected and these are deposited in herbaria awaiting identification (Bebber et al., 2010). On the other hand, Fontaine, Perrard & Bouchet (2012) calculated that an average time between the first collection of a specimen of a new species to its formal description is 21 years. Considering the ongoing habitat loss it is not therefore surprising that numerous novelties found in herbarium are difficult to locate in its natural habitat and for that reason the number of samples used in new species description is often limited. According International Plant Name Index (IPNI) only in 2019 more than 150 new Orchidaceae species were described. Novelties were found mainly in tropical in subtropical regions and numerous new names, for example *Dichaea amazonica* Pupulin, *Epidendrum brevicallosum* Hágsater & E. Santiago, were published based on single specimen. By examination of numerous *Pterichis* from various geographical regions (Kolanowska & Olędrzyńska, 2015; Kolanowska & Szlachetko, 2017) we were able to recognize the diagnostic characters of this genus representatives and only these taxonomically important traits were used to describe new species.

The last study which included the recognition of *Pterichis* diversity in Ecuador included only six species (Garay, 1978). In contrast to Garay’s (1978) concept which did not recognize *P. seleniglossa* as separated species, in our opinion this taxon clearly differs from all other genus representatives and can be distinguished from similar *P. triloba* by 1-veined petals (vs. 3-veined). In our study we did not find any population of *P. triloba* or *P. seleniglossa* with intermediate, 2-veined petals hereby in our opinion this character is sufficient for species separateness.

## Conclusions

Here we presented the synopsis of Ecuadorian representatives of *Pterichis*. In our opinion species characteristics and identification keys will be useful for local scientists working on local floras inventories and for launching more sophisticated nature management programs.

We confirmed the occurrence of 17 *Pterichis* species in Ecuador. That number includes four new species described in this article. Seven genus representatives are endemic and were not so far found outside the country—*P. ansaloniana, P. dodsoniana, P. elliptica, P hirtziana, P. madsenii, P. meirax*, and *P. tunguraguona*. National *Pterichis* occur in just three ecoregions (Eastern Cordillera real montane forests, Northern Andean páramo, Northwestern Andean montane forests) and they are growing usually as terrestrial herbs between 2,300 and 4,110 m a.s.l.

## Additional Information and Declarations

### Data Availability

The following information was supplied regarding data availability: This is taxonomic revision; all data (e.g., list of herbairum specimens examined) are presented in the article in the sections entitled “Representative Specimens” under characteristic of each taxon. New species are deposited: Pterichis ansaloniana—C. Ulloa et al. 1361 (HA 5333) Pterichis madsenii—J.E. Madsen & L. Ellemann 75293 (AAU s.n.). Pterichis hirtziana—H. van der Werff & W. Palacios 8956 (QCNE 34289). Pterichis dodsoniana—L.B. Holm-Nielsen et al. 4767 (AAU s.n.).

### New Species Registration

The following information was supplied regarding the registration of a newly described species: Pterichis ansaloniana Kolan., Szlach. & S. Nowak, sp. nov.: 77214449-1 Pterichis madsenii Kolan., Szlach. & S. Nowak, sp. nov.: 77214450-1 Pterichis hirtziana Kolan., Szlach. & S. Nowak, sp. nov.: 77214451-1 Pterichis dodsoniana Kolan., Szlach. & S. Nowak, sp. nov.: 77214452-1 Pterichis elliptica var. ecuadorensis Kolan., Szlach. & S. Nowak, var. nov.: 77214453-1 *Pterichis multiflora* var. ecuadorensis Kolan., Szlach. & S. Nowak, var. nov.: 77214454-1
